# Pan-cancer analysis of CREB3L1 as biomarker in the prediction of prognosis and immunotherapeutic efficacy

**DOI:** 10.3389/fgene.2022.938510

**Published:** 2022-09-09

**Authors:** Zhengjun Lin, Yanlin Wu, XunGang Xiao, Xianghong Zhang, Jia Wan, Tao Zheng, Hongxuan Chen, Tang Liu, Xianzhe Tang

**Affiliations:** ^1^ Department of Orthopedics, The Second Xiangya Hospital, Central South University, Changsha, Hunan, China; ^2^ Department of Orthopedics, Chenzhou No. 1 People’s Hospital, Chenzhou, Hunan, China

**Keywords:** CREB3L1, pan-cancer, prognostic biomarker, tumor immune microenvironment, immunotherapy efficacy, drug sensitivity

## Abstract

**Background:** CAMP response element binding protein 3-like 1 (CREB3L1) has been indicated as a critical biomarker and can modulate multifaced behaviors of tumor cells in diverse cancers. However, a systematic assessment of CREB3L1 in pan-cancer is of absence, and the predictive value of CREB3L1 in cancer prognosis, the tumor immune microenvironment and the efficacy of immunotherapy remains unexplored.

**Methods:** CREB3L1 expression in 33 different cancer types was investigated using RNAseq data from The Cancer Genome Atlas (TCGA) database. The characteristics of CREB3L1 alternations were illustrated in cBioPortal database. The prognostic and clinicopathological value of CREB3L1 was analyzed through clinical data downloaded from the TCGA database. The potential role of CREB3L1 in the tumor immune microenvironment was illustrated by utilizing CIBERSORT and ESTIMATE algorithms, and TISIDB online database. The associations between CREB3L1 expression and tumor mutation burden (TMB), and microsatellite instability (MSI) were assessed by spearman’s rank correlation coefficient. Furthermore, Gene Set Enrichment Analysis (GSEA) was conducted to explore the potential biological functions and downstream pathways of CREB3L1 in different human cancers. The correlations of CREB3L1 expression with PD-1/PD-L1 inhibitors efficacy and drug sensitivity were also investigated.

**Results:** The expression of CREB3L1 was abnormally high or low in several different cancer types, and was also strictly associated with the prognosis of cancer patients. CREB3L1 expression levels have a strong relationship with infiltrating immune cells, including regulatory T cells, CD8^+^ T cells, macrophages, B naïve cells, dendritic cells and mast cells. CREB3L1 expression was also correlated with the expression of multiple immune-related biomolecules, TMB, and MSI in several cancers. Moreover, CREB3L1 had promising applications in predicting the immunotherapeutic benefits and drug sensitivity in cancer management.

**Conclusions:** Our results highlight the value of CREB3L1 as a predictive biomarker for the prognosis and immunotherapy efficacy in multiple cancers, and CREB3L1 seems to play key roles in the tumor immune microenvironment, suggesting the role of CREB3L1 as a promising biomarker for predicting the prognosis and immune-related signatures in diverse cancers.

## Introduction

In the past decades, advances in cancer immunotherapy have evidently improved the therapeutic efficacy in the treatment of diverse cancers via the interactions between the human immune system and tumor (Yang, 2015; Kennedy and Salama, 2020). Despite immunotherapies have been successfully applied across a wide range of human malignancies, the majority of cancer patients possess limited or no responses to immunotherapies (Sharma et al., 2017; O'Donnell et al., 2019). Emerging evidence has confirmed that the tumor immune microenvironment, such as infiltrating immune cells, and the expression of immune checkpoints is critically modulated by multiple biomolecules, which may significantly influence the efficacy of immunotherapy (Lei et al., 2020; Kumagai et al., 2021). Hence, developing a promising biomarker to predict the tumor immune microenvironment-related signatures and immunotherapeutic responses of cancer patients at early stage is warranted.

The CREB3 family of transcription factors are endoplasmic reticulum (ER) localized proteins and belong to the large bZIP family (Sampieri et al., 2019). CREB3 family members play important roles in modulating tissue development, lipids metabolism, proteins secretion, cell differentiation, and tumorigenesis (Sampieri et al., 2019). Notably, recent studies have reported CREB3L1, a critical member of CREB3 family, participate in cancer initiation and progression, and can serve as a promising clinical biomarker for cancer patients (Vellanki et al., 2013; Sampieri et al., 2019; Morishita et al., 2021). For instance, it has been found that the combination of doxorubicin and oncolytic vaccinia virus can induce cell death through the activation of CREB3L1 in ovarian cancer (Mistarz et al., 2021). Several studies have indicated that CREB3L1 may function as the suppressor of cellular metastasis and proliferation through a similar mechanism to p53 (Denard et al., 2011). Mellor et al. have reported that CREB3L1 is a critical tumor suppressor that can inhibit metastasis, invasion, and angiogenesis *in vitro* (Mellor et al., 2013). On the contrary, several studies have reported opposite conclusions that CREB3L1 can contribute to cancer cells metastasis and invasion. CREB3L1 expression is significantly upregulated in distant metastasis of triple-negative breast cancers (TNBC) patients. Besides, cancer-specific PERK signaling induces cell invasion and metastasis through directly targeting CREB3L1 in breast cancer through inducing epithelial-mesenchymal transition (EMT) by an ATF4-Fra-1 interaction. Mechanistically, CREB3L1 can promote breast cancer cell invasion and metastasis through inducing ECM production by activating FAK, and a chemical inhibitor of proteases, AEBSF can inhibit breast cancer cell invasion by inhibiting CREB3L1 expression (Feng et al., 2017). Furthermore, it has been identified that CREB3L1 can function as a prognostic and diagnostic biomarker, and predict the chemotherapeutic responses in some cancer types (Denard et al., 2018; Liu et al., 2018; Morishita et al., 2021). These results indicate the critical roles of CREB3L1 in cancer development, however, different studies present controversial results which need to be clarified. Moreover, the role of CREB3L1 in modulating the tumor immune microenvironment is still largely unknown, and no systematic pan-cancer investigation has focused on CREB3L1.

In this pan-cancer analysis research, we analyzed the expression pattern of CREB3L1 and its relationship with the prognosis of cancer patients. Furthermore, we explored the relationship between CREB3L1 and the immune cells infiltration, the expression of immune-related biomarkers, and immunotherapeutic efficacy. Our study sheds highlight on the promising role of CREB3L1 as a prognostic biomarker, and an effective predictor for the immunotherapeutic efficacy and drug sensitivity in pan-cancer, as well as the potential mechanism by which CREB3L1 modulates the tumor immune microenvironment.

## Materials and methods

### Data acquisition

RNA sequencing (RNA-seq) data, somatic mutation, and related clinical data of 33 cancer types were downloaded from TCGA dataset, and RNA-seq data of 54 normal tissue samples in Genotype-Tissue Expression (GTEx) dataset were extracted from UCSC Xena (https://xena.ucsc.edu/) (Goldman et al., 2015). The Strawberry Perl software (Version 5.32.1.1, http://strawberryperl.com/) was used to extract the CREB3L1 expression data from these downloaded datasets, each expression value was normalized by log2 transformation. Cellular CREB3L1 expression data in 24 different cancer cell lines were downloaded from the Broad Institute Cancer Cell Line Encyclopedia (CCLE) portal (https://portals.broadinstitute.org/ccle/about) (Barretina et al., 2012). The differential CREB3L1 expression between diverse cancer types and their corresponding normal samples were evaluated by student *t*-test and visualized via R package “ggplot2”.

### Genetic alternation analysis

Genetic alteration analysis of CREB3L1 was performed based on the “TCGA Pan-Cancer Atlas Studies” dataset in the cBioPortal (https://www.cbioportal.org), a dataset that includes diverse data types, such as DNA methylation data, transcription data, non−synonymous mutations, and DNA copy number data (Gao et al., 2013). We applied the data to investigate the mutation rate and characteristics of CREB3L1 alterations in pan-cancer.

### Prognostic analysis

Survival and clinical phenotype data were acquired from the TCGA dataset. The Kaplan-Meier analysis and the univariate Cox regression analyses were performed to comprehensively investigate the relationship between the CREB3L1 expression levels and the prognosis of patients in pan-cancer, including overall survival (OS), progression-free survival (PFS), disease-free survival (DFS), and disease-specific survival (DSS) in pan-cancer by R-packages “survival”, “survminer”, “forestplot”, “limma” and “ggpubr”.

### Relationship between CREB3L1 expression and immunity

Estimation of Stromal and Immune cells in Malignant Tumor tissues using Expression data (ESTIMATE), a bioinformatics algorithm that infers the degree of infiltration of immune cells and stromal cells in tumor samples was exploited to evaluate the stroma and immune scores of each sample using existing CREB3L1 expression data in pan-cancer via the R package “estimate” (Yoshihara et al., 2013). CIBERSORT, a bioinformatics algorithm that quantifies the cellular composition of tissue samples by their gene expression, was performed to calculate the correlation coefficient for diverse immune cells in 32 different cancer types (Chen et al., 2018). Associations between the immune cell infiltration degree and CREB3L1 expression in pan-cancer were investigated by R-packages “ggExtra”, “ggplot2” and “ggpubr”. The immune cells infiltration in pan-cancer was also evaluated utilizing MCPcounter (Dienstmann et al., 2019), XCELL (Aran, 2020), TIMER (Li et al., 2020), QUANTISEQ (Plattner et al., 2020), EPIC (Racle et al., 2017), and IPS (Charoentong et al., 2017) algorithms in Sangerbox (http://www.sangerbox.com/tool). Furthermore, we investigated the correlation of CREB3L1 expression with the expression of immune-related biomolecules, and immune molecular subtypes in the TISIDB database (http://cis.hku.hk/TISIDB/index.php). The TISIDB database is an online portal integrating abundant data types, such as high-throughput screening and RNA-seq data from the TCGA database for analyzing the tumor and immune system interaction (Ru et al., 2019).

### Gene set enrichment analysis

To explore the underlying biological functions and signaling pathways of CREB3L1 in pan-cancer, we subsequently performed the GSEA. Kyoto Encyclopedia of Genes and Genomes (KEGG) and Gene ontology (GO) gene database were extracted from the GSEA website (https://www.gsea-msigdb.org/gsea/downloads.jsp). GSEA was conducted utilizing the R packages “limma”, “org.Hs.eg.db”, “enrichplot” and “clusterProfiler” with the following parameters: nPerm = 100, and p-value-Cutoff = 1.

### Correlation of CREB3L1 expression with TMB and MSI

The TMB and MSI scores were downloaded from TCGA pan-cancer mutation data (https://tcga.xenahubs.net). TMB, which represents the alteration frequency in a distinct cancer type, is a quantifiable biomarker assessing the immunotherapeutic response of PD-1 antibodies (Chan et al., 2019). MSI is a new microsatellite allele in a tumor due to a deficient mismatch repair system of DNA, and has been recognized as a critical tumor biomarker (Vilar and Gruber, 2010). A Perl script were performed to calculate TMB scores and revised by dividing the total length of exons. The correlation analysis between the CREB3L1 expression levels and TMB or MSI was elaborated using Spearman’s rank correlation coefficient. R-package “fmsb” was used to draw Radar plots.

### Relationship between CREB3L1 expression and the efficacy of immunotherapy

To evaluate the correlation between CREB3L1 expression and the efficacy of immunotherapy, we enrolled three databases including GSE78220 (melanoma) (Hugo et al., 2016), GSE67501 (renal cell carcinoma) (Ascierto et al., 2016), and IMvigor210 (metastatic urothelial cancer), which were downloaded from GEO (https://www.ncbi.nlm.nih.gov/geo/) (Charoentong et al., 2017). The procedure was conducted and the results were visualized by utilizing R-package “ggpubr” and “ggplot2”.

### Relationship between CREB3L1 expression and drug sensitivity

To elucidate the correlation between CREB3LI expression with drug sensitivity in pan-cancer, NCI-60 compound activity data with RNA-seq expression profiles were downloaded from the CallMiner™ online database (https://discover.nci.nih.gov/cellminer/home.do). The correlation between CREB3LI expression and the sensitivity of drugs approved by FDA was analyzed by utilizing R packages “impute”, “limma”, “ggplot2”, and “ggpubr”.

### Statistical analysis

Student’s *t*-test was used to analyze the differences on gene expression data between groups. For survival analyses, the Kaplan-Meier curve and Cox analysis were performed. Statistical analyses and visualization were performed on R software (Version 4.1.2, https://www.Rproject.org). *p* < 0.05 was indicated the statistical significance (*p < 0.05, **p < 0.01, ***p < 0.001, and ****p < 0.0001).

## Results

### CREB3L1 expression analysis in pan-cancer

**FIGURE 1:**
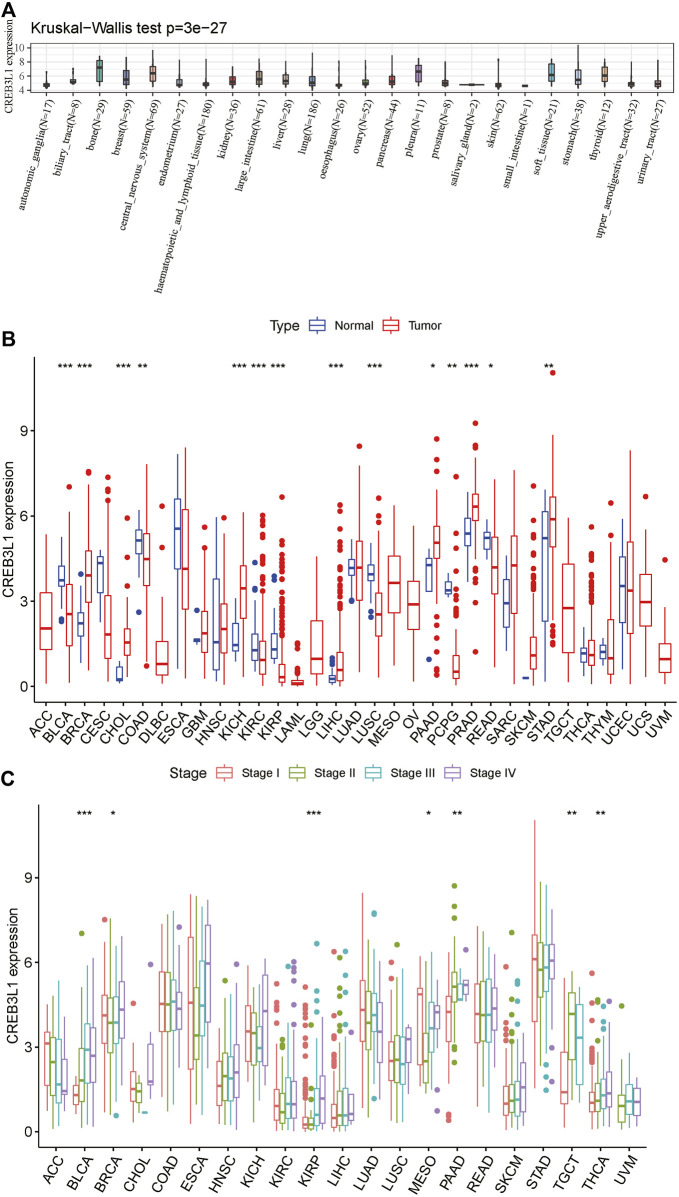
The expression pattern of CREB3L1. **(A)** CREB3L1 expression in 24 cancer cell lines from CCLE database. **(B)** Comparison of CREB3L1 expression between cancer and matched normal samples from TCGA database. **(C)** CREB3L1 expression in patients with different WHO stages in pan-cancer from TCGA database. *p < 0.05, **p < 0.01, ***p < 0.001.

To start with, we explored CREB3L1 expression in diverse cancer cell lines in the CCLE database. The results showed that CREB3L1 expression varied from different cancer cell lines, and the expression levels of CREB3L1 were highest in bone, pleura and central nervous system cancer cell lines, while CREB3L1 was expressed lowly in small intestine, oesophagus and salivary gland cancer cell lines compared with other cancer cell lines (Figure 1A). Next, we compared CREB3L1 expression between normal and tumor samples in TCGA pan-cancer database, and we detected significant differences in 14 cancer types among 33 types of cancer excluding those without normal tissue data (Wilcoxon test *p* < 0.05) (Figure 1B). The results revealed that significantly higher CREB3L1 expression was observed in 7 cancer types than their corresponding adjacent non-cancerous tissues, including breast invasive carcinoma (BRCA), cholangiocarcinoma (CHOL), kidney chromophobe (KICH), liver hepatocellular carcinoma (LIHC), pancreatic adenocarcinoma (PAAD), prostate adenocarcinoma (PRAD) and stomach adenocarcinoma (STAD). In comparison, CREB3L1 expression levels were abnormally downregulated in tumor samples in 7 cancer types, including bladder urothelial carcinoma (BLCA), colon adenocarcinoma (COAD), kidney renal clear cell carcinoma (KIRC), kidney renal papillary cell carcinoma (KIRP), lung squamous cell carcinoma (LUSC), pheochromocytoma and paraganglioma (PCPG) and rectum adenocarcinoma (READ). To further illustrate the clinical significance of CREB3L1 in pan-cancer, we also assessed the CREB3L1 expression levels in different cancer clinical stages, and the results suggested that CREB3L1 expression was significantly related to the clinical stage in 7 cancer types, in which CREB3L1 expression levels were relatively higher in later clinical stages, including BLCA, BRCA, KIRP, mesothelioma (MESO), PAAD, testicular germ cell tumor (TGCT), thyroid carcinoma (THCA), while in other 14 caner types, CREB3L1 expression was not significantly modified during clinical stage progression (Figure 1C). Interestingly, CREB3L1 expression levels were higher in patients with stage I MESO and decreased in patients with stage II MESO. Then, the expression of CREB3L1 was gradually increased in stage III/IV MESO patients. Despite the increasing trend, CREB3L1 expression in stage IV MESO patients was still lower than those in stage I MESO patients. In addition, we also analyzed physiologic CREB3L1 gene expression levels in normal tissues based on the GTEx database. The results showed that CREB3L1 was highly expressed in the salivary gland, prostate and stomach tissues, while was lowly expressed in the blood, liver and muscle tissues in comparison with other normal tissues (Supplementary Figure S1A). We also evaluated CREB3L1 expression in pan-cancer, which was ranked from high to low (Supplementary Figure S1B). We found that CREB3L1 was expressed in 33 cancer types, in detail, CREB3L1 was expressed highest in PRAD while the lowest was in acute myeloid leukemia (LAML). Then, we also explored the differential CREB3L1 mRNA expression between cancer samples in TCGA and normal tissue samples in GTEx. A significant difference of CREB3L1 expression was detected in 25 cancer types (Supplementary Figure S1C). The results were basically consistent with the differential expression analysis in TCGA pan-cancer cohorts. In addition to cancer types mentioned above, the results showed that CREB3L1 was also abnormally upregulated in glioblastoma multiforme (GBM), lower grade glioma (LGG), lung adenocarcinoma (LUAD), ovarian serous cystadenocarcinoma (OV), TGCT, and uterine carcinosarcoma (UCS), whereas was abnormally downregulated in adrenocortical carcinoma (ACC), cervical squamous cell carcinoma (CESC), LAML, skin cutaneous melanoma (SKCM), and THCA. But in COAD, CREB3L1 expression was significantly upregulated in tumor samples compare with corresponding normal samples in GTEx, which was contrary to the result presented in Figure 1B.

### Genetic alteration analysis of CREB3L1 in different cancers

**FIGURE 2:**
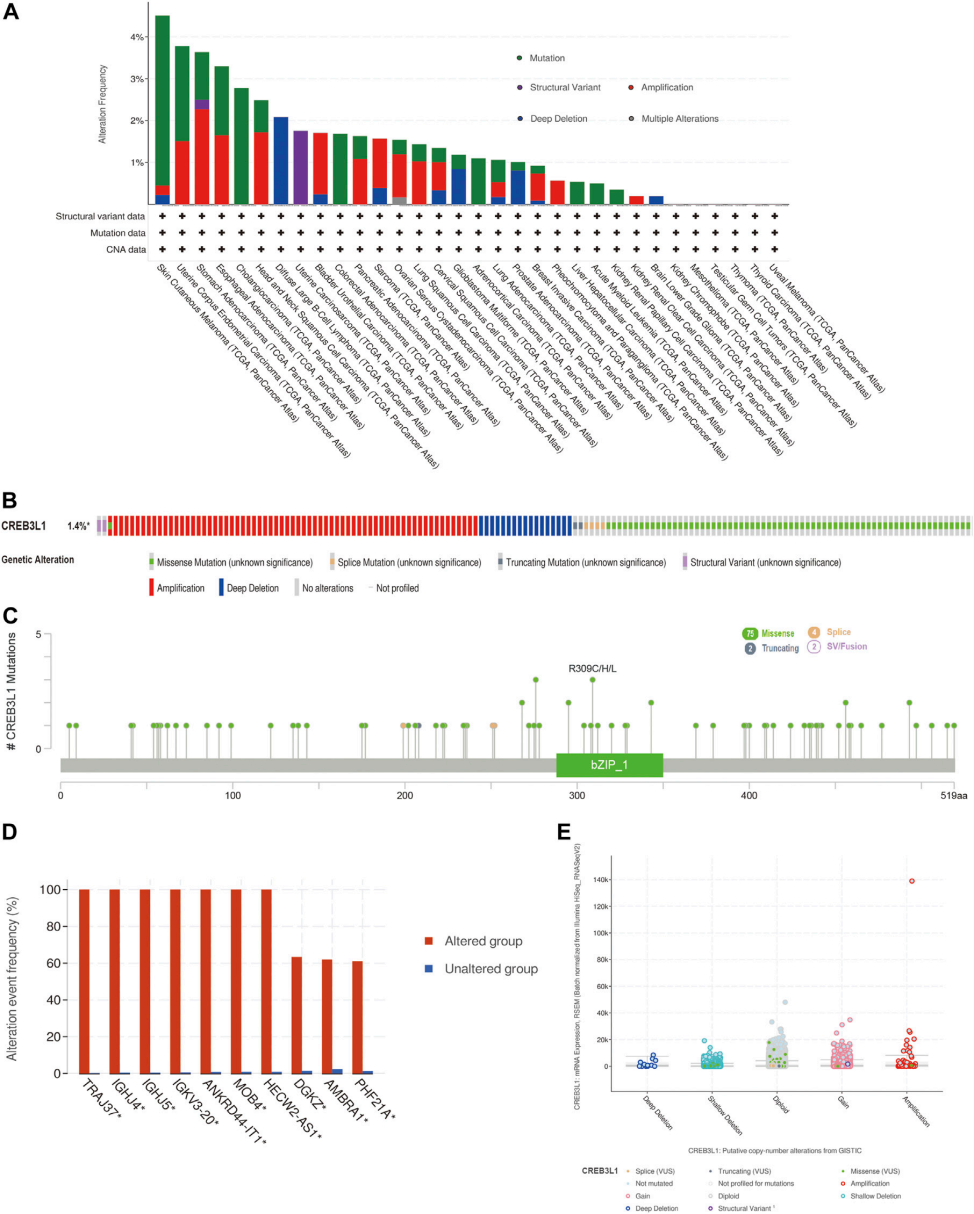
The genetic alteration characteristics of CREB3L1 in pan-cancer. **(A)** The alteration frequency of CREB3L1 with different types of mutations in various cancer types. **(B)** Summary of different genetic alteration types of CREB3L1 (Different colors refers to different types of CREB3L1 genetic alterations). **(C)** The mutation types, sites, and sample numbers of the CREB3L1 genetic alterations. **(D)** Co-occurrence of genetic mutations in tumors with CREB3L1 alterations. **(E)** The correlated alteration types and putative copy-number of CREB3L1 in pan-cancer. * Altered genes.

We further explored the characteristics of CREB3L1 genetic alternations in different cancers by enrolling the TCGA Pan-Cancer Atlas studies in cBioPortal database. The results found that CREB3L1 was altered in 158 patients (1.4%) of 10953 patients. In patients with SKCM, uterine corpus endometrial carcinoma (UCEC), esophageal adenocarcinoma (ESCA), CHOL, and head and neck squamous cell carcinoma (HNSC), the high CREB3L1 gene alteration rate was occurred, in which the alteration frequency was higher than 2% (Figure 2A). The primary genetic alteration types of CREB3L1 were amplification, miss mutation, and deep deletion (Figure 2B). As shown in Figure 2C, the mutation sites, types, and sample numbers of the CREB3L1 genetic alterations were presented. In addition, the main type alteration was CREB3L1 missense mutation, while R309C alteration was detected in LUSC, R309H alteration was detected in uterine papillary serous carcinoma and R309L alteration was detected in KIRP. Genomic alternations co-occurrence analysis indicated that alterations of several genes, including TRAJ37, IGHJ4, IGHJ5, IGKV3-20, ANKRD44-IT1, MOB4, HECW2-AS1, DGKZ, AMBRA1, and PHF21A were more commonly occurred in the CREB3L1-altered group (Figure 2D). Besides, the putative copy-number alterations of CREB3L1 from genomic identification of significant targets in cancer (GISTIC) included many types, such as amplification, deep deletion and gain function, resulting in the alternations of gene expression (Figure 2E).

### Prognostic significance of CREB3L1

**FIGURE 3:**
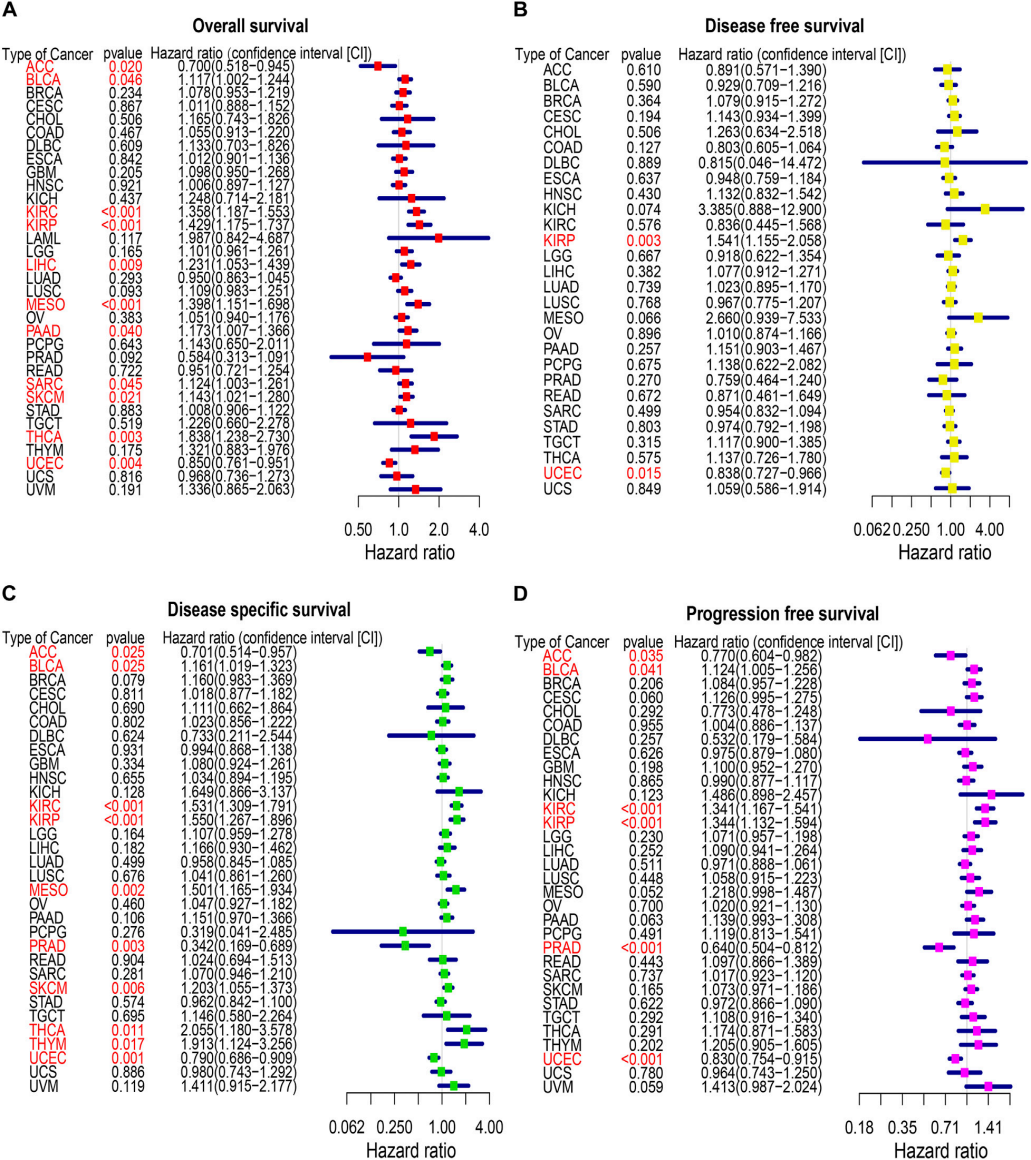
The forest map of univariate Cox regression analysis of CREB3L1. **(A)** Forest map shows the results of univariate cox regression analysis of CREB3L1 for OS in TCGA pan-cancer. **(B)** Forest map shows the results of univariate cox regression analysis of CREB3L1 for DFS in TCGA pan-cancer. **(C)** Forest map shows the results of univariate cox regression analysis of CREB3L1 for DSS in TCGA pan-cancer. **(D)** Forest map shows the results of univariate cox regression analysis of CREB3L1 for PFS in TCGA pan-cancer. Red items indicate the statistical significance.

To identify the prognostic significance of CREB3L1 in cancer patients, we further performed a comprehensive prognosis analysis using the survival data of TCGA pan-cancer dataset, including OS, DFS, DSS and PFS. The univariate Cox regression analysis were performed and found CREB3L1 expression levels were correlated with OS in 11 cancers, DFS in two cancers, DSS in 10 cancers, and PFS in six cancers (Figures 3A–D). The results of univariate Cox regression analysis showed that the CREB3L1 expression levels were strongly related to OS in ACC (hazard ratio [HR], 0.700; 95% confidence interval [CI], 0.518-0.945; *p* = 0.020), BLCA (HR, 1.117; 95% CI, 1.002-1.244; *p* = 0.046), KIRC (HR, 1.358; 95% CI, 1.187-1.553; *p* < 0.001), KIRP (HR, 1.429; 95% CI, 1.175-1.737; *p* < 0.001), LIHC (HR, 1.231; 95% CI, 1.053-1.439; *p* = 0.009), MESO (HR, 1.398; 95% CI, 1.151-1.698; *p* < 0.001), PAAD (HR, 1.173; 95% CI, 1.007-1.366; *p* = 0.040), sarcoma (SARC) (HR, 1.124; 95% CI, 1.003-1.261; *p* = 0.045), SKCM (HR, 1.143; 95% CI, 1.021-1.280; *p* = 0.021), THCA (HR, 1.838; 95% CI, 1.238-2.730; *p* = 0.003) and UCEC (HR, 0.850; 95% CI, 0.761-0.951; *p* = 0.004) (Figure 3A). In the results, we found that CREB3L1 was a risk factor in BLCA, KIRC, KIRP, LIHC, SARC, SKCM and THCA, while it was a protective factor in ACC and UCEC. The DFS analysis revealed that CREB3L1 could act as a protective factor for patients with UCEC (HR, 0.838; 95% CI, 0.727-0.966; *p* = 0.015), while a risk factor for patients with KIRP (HR, 1.541; 95% CI, 1.155-2.058; *p* = 0.003) (Figure 3B). The DSS analysis indicated that CREB3L1 serves as a protective factor for patients with ACC (HR, 0.701; 95% CI, 0.514-0.957; *p* = 0.025), PRAD (HR, 0.342; 95% CI, 0.169-0.689; *p* = 0.003) and UCEC (HR, 0.790; 95% CI, 0.686-0.909; *p* = 0.001), and acts as a risk role in patients with BLCA (HR, 1.161; 95% CI, 1.019-1.323; *p* = 0.025), KIRC (HR, 1.531; 95% CI, 1.309-1.791; *p* < 0.001), KIRP (HR, 1.550; 95% CI, 1.267-1.896; *p* < 0.001), MESO (HR, 1.501; 95% CI, 1.165-1.934; *p* = 0.002), SKCM (HR, 1.203; 95% CI, 1.055-1.373; *p* = 0.006), THCA (HR, 2.055; 95% CI, 1.180-3.578; *p* = 0.011) and thymoma (THYM) (HR, 1.913; 95% CI, 1.124-3.256; *p* = 0.017) (Figure 3C). In addition, the PFS analysis suggested that CREB3L1 plays a protective role in patients with ACC (HR, 0.770; 95% CI, 0.604-0.982; *p* = 0.035), PRAD (HR, 0.640; 95% CI, 0.504-0.812; *p* < 0.001) and UCEC (HR, 0.830; 95% CI, 0.754-0.915; *p* < 0.001) and a risk role in patients with BLCA (HR, 1.124; 95% CI, 1.005-1.256; *p* = 0.041), KIRC (HR, 1.341; 95% CI, 1.167-1.541; *p* < 0.001) and KIRP (HR, 1.344; 95% CI, 1.132-1.594; *p* < 0.001) (Figure 3D).

**FIGURE 4:**
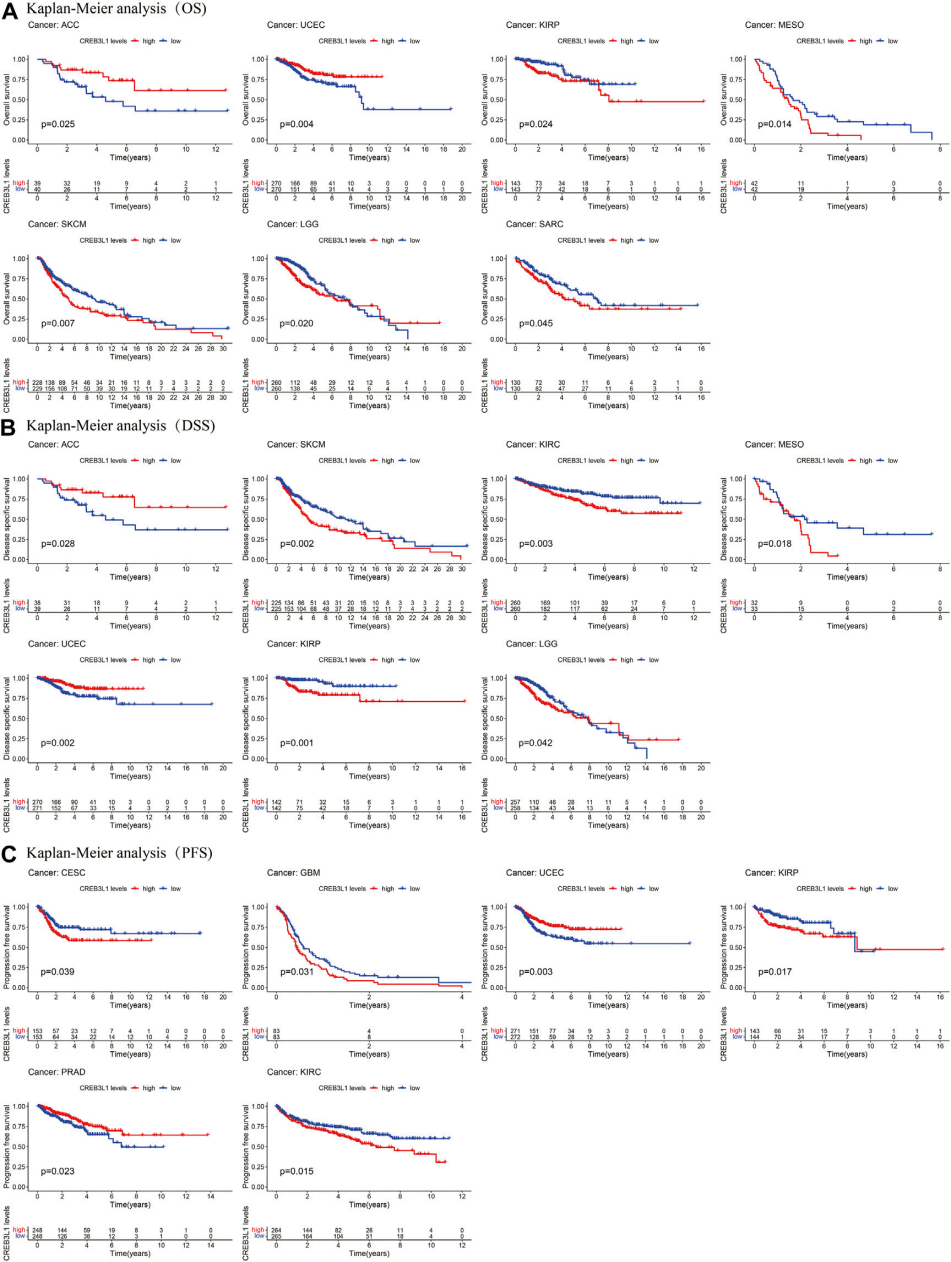
Kaplan-Meier survival curves of CREB3L1 in pan-cancer. **(A)** Kaplan–Meier analysis of the correlation between CREB3L1 expression and OS in 7 cancer types. **(B)** Kaplan–Meier analysis of the correlation between CREB3L1 expression and DSS in 7 cancer types. **(C)** Kaplan–Meier analysis of the correlation between CREB3L1 expression and PFS in six cancer types.

The Kaplan-Meier analysis indicated that the CREB3L1 expression levels were correlated with OS in 7 cancers, DSS in 7 cancers, and PFS in six cancers (Figures 4A–C). Our results of Kaplan-Meier OS analysis demonstrated that among patients with ACC (*p* = 0.025) and UCEC (*p* = 0.004), longer survival time was presented in those who with high levels of CREB3L1 expression, however, in patients with KIRP (*p* = 0.024), MESO (*p* = 0.014), SKCM (*p* = 0.007), LGG (*p* = 0.020) and SARC (*p* = 0.045), high CREB3L1 expression levels were associated with poor OS (Figure 4A). The analysis of DSS revealed correlations between high CREB3L1 expression levels and good prognosis in patients with ACC (*p* = 0.028), UCEC (*p* = 0.002) and LGG (*p* = 0.042), which was opposite in patients with SKCM (*p* = 0.002), KIRC (*p* = 0.003), MESO (*p* = 0.018) and KIRP (*p* = 0.001) (Figure 4B). The Kaplan-Meier curves of PFS indicated that in UCEC (*p* = 0.003) and PRAD (*p* = 0.023), patients with low CREB3L1 expression levels had a shorter survival time, while in CESC (*p* = 0.039), GBM (*p* = 0.031), KIRP (*p* = 0.017) and KIRC (*p* = 0.015), there were better prognoses in patients with low CREB3L1 expression levels (Figure 4C). These results indicated that CREB3L1 expression levels were significantly associated with cancer prognosis, and might exert different effects on prognoses in different cancer types.

### Correlation of CREB3L1 expression with the tumor immune microenvironment

**FIGURE 5:**
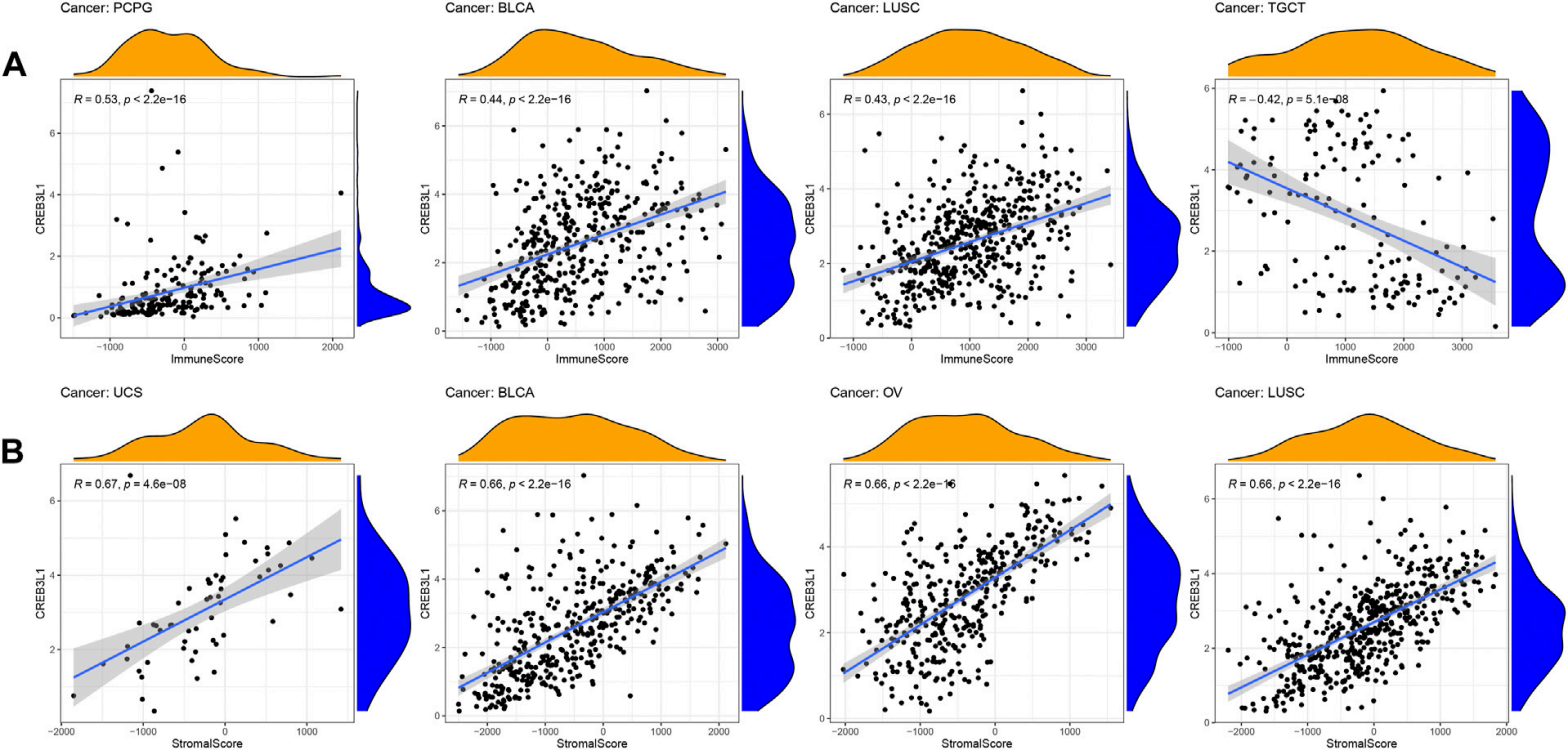
The correlation between CREB3L1 and immune and stromal scores in pan-cancer. **(A)** The association between CREB3L1 expression and immune score in PCPG, BLCA, LUSC, and TGCT. **(B)** The association between CREB3L1 expression and stromal score in top 4 cancer types, including UCS, BLCA, OV, and LUSC.

The tumor immune microenvironment plays important roles in tumor initiation and progression. Therefore, exploring the relationship between CREB3L1 expression and the tumor immune microenvironment in pan-cancer is vital. To calculate the stromal and immune scores in pan-cancer, and explore the association between these two scores and CREB3L1 expression levels, the ESTIMATE algorithm was employed for research. The results showed that CREB3L1 expression levels were positively correlated with immune scores in BLCA, LUSC, and PCPG, whereas were negatively related to immune scores in TGCT (Figure 5A). In addition, there was positive relationship between CREB3L1 expression and stromal scores in multiple cancers, particularly in UCS, BLCA, OV, and LUSC (Figure 5B). The relationship between CREB3L1 expression levels and stromal scores in other cancer types was shown in Supplementary Figure S2.

**FIGURE 6:**
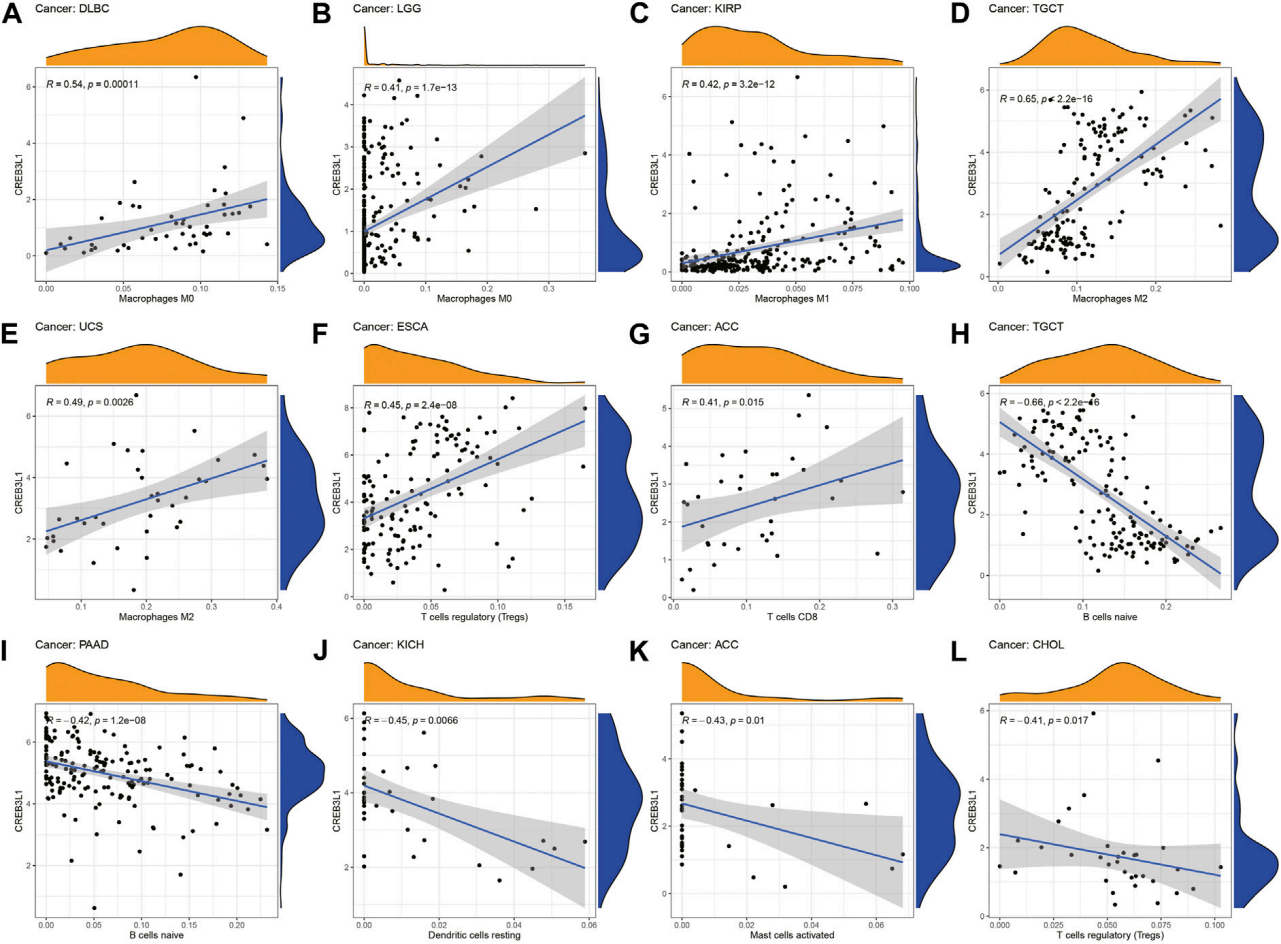
The correlation between CREB3L1 expression and the immune cells infiltration in pan-cancer. **(A–L)** CREB3L1 expression was associated with macrophages, regulatory T cells, CD8 + T cells, B cells naïve, dendritic cells and mast cells in different cancer types.

We further explored the relationship between CREB3L1 expression levels and the infiltration levels of 22 relevant immune cells. The results indicated that CREB3L1 expression levels were strongly related to tumor immune cell infiltration levels in several cancer types. We detected that CREB3L1 expression was positively associated with the infiltration levels of macrophages in several cancer types, including macrophage M0 in DLBC (Figure 6A) and LGG (Figure 6B), macrophage M1 in KIRP (Figure 6C), macrophage M2 in TGCT (Figure 6D) and UCS (Figure 6E), and regulatory T cells in ESCA (Figure 6F), CD8^+^ T cells in ACC (Figure 6G), while was negatively associated with the abundance of B cells naïve in TGCT (Figure 6H) and PAAD (Figure 6I), dendritic cells resting in KICH (Figure 6J), mast cells activated in ACC (Figure 6K) and regulatory T cells in CHOL (Figure 6L). Furthermore, we also used the XCELL, TIMER, QUANTISEQ, EPIC, IPS and MCPcounter databases to analyze the association between CREB3L1 expression and the infiltrating levels of immune cells in pan-cancer (Supplementary Figure S3).

**FIGURE 7:**
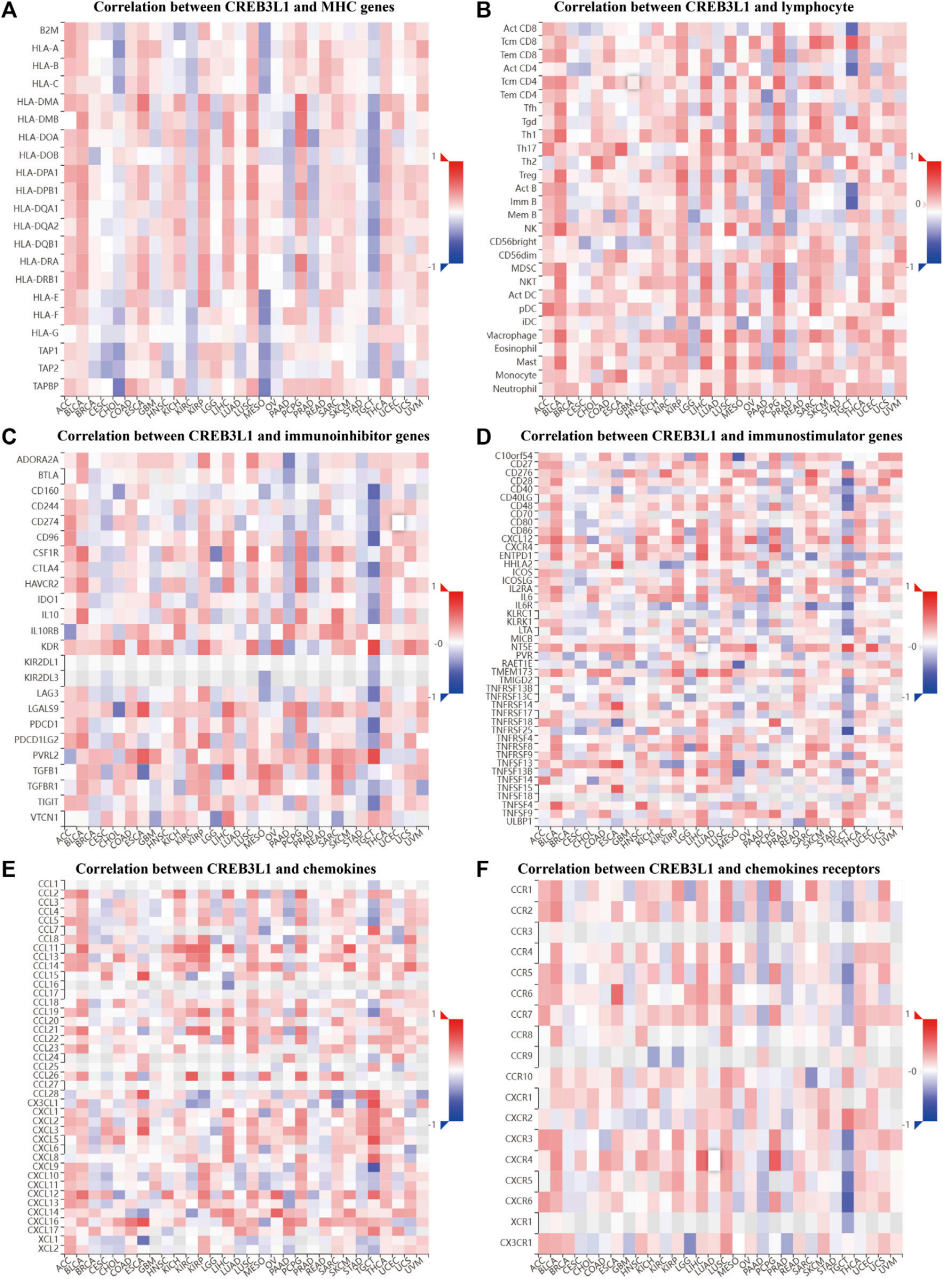
The correlation between CREB3L1 expression and immune-related biomarkers in TISIDB database. The co-expression heatmaps show the association between CREB3L1 expression and **(A)** MHC genes, **(B)** lymphocyte, **(C)** immunoinhibitor genes, **(D)** immunostimulator genes, **(E)** chemokines, and **(F)** chemokines receptors in different cancers.

To further explore the potential function of CREB3L1 in regulating the tumor immune microenvironment in human cancers, we examined the associations between CREB3L1 expression and the expression of major histocompatibility complex (MHC) genes (Figure 7A), lymphocyte (Figure 7B), immunoinhibitor genes (Figure 7C), immunostimulator genes (Figure 7D), chemokine (Figure 7E) and chemokine receptors (Figure 7F) by gene co-expression analyses in TISIDB. Our study revealed that CREB3L1 expression was distinctly correlated with multiple immuneinhibitors genes, such as KDR, PVRL2, CD160, and CD96, and immunostimulators genes, such as NT5E, TNFRSF14, CD28, and CXCR4, and chemokines including CX3CL1, CXCL9, CCL15, and CXCL5 in multiple cancer types, suggesting the interactions between CREB3L1 and immune-related biomarkers expression in pan-cancer.

### Gene set enrichment analysis

**FIGURE 8:**
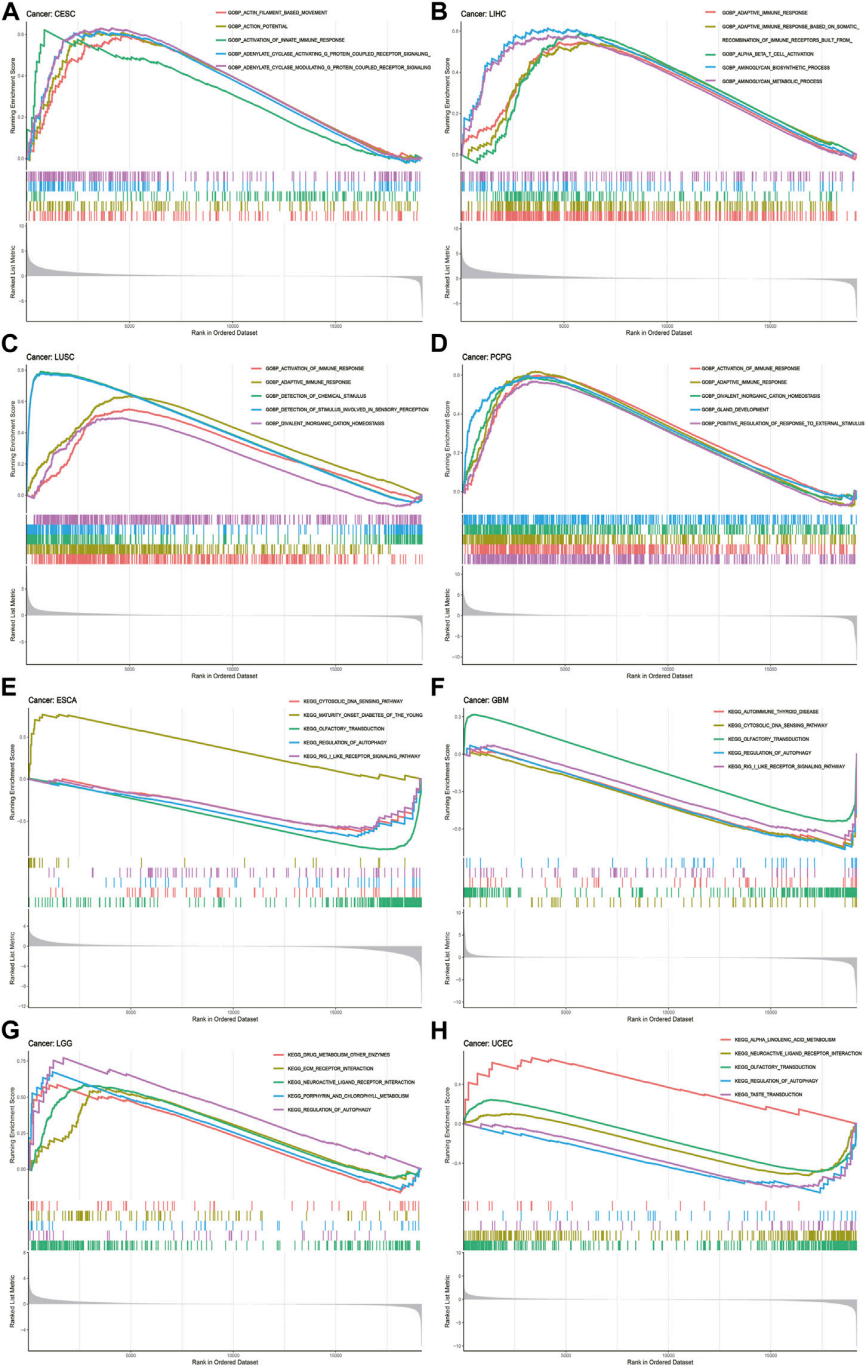
GSEA of CREB3L1. **(A–D)** GO functional annotation of CREB3L1 shows the activation of innate immune response in CESC, and adaptive immune response in LIHC, LUSC, and PCPG. **(E–H)** KEGG pathway analysis of CREB3L1 shows that regulation of autophagy was the most common signaling pathway of CREB3L1 participating in TCGA pan-cancer, including UCEC, LGG, GBM, and ESCA. Curves of different colors show different functions or pathways regulated in different cancers. Peaks on the upward curve indicate positive regulation and peaks on the downward curve indicate negative regulation.

To deeply uncover the potential biological functions and signaling pathways of CREB3L1 in pan-cancer, GESA, including GO functional annotation and KEGG pathway analysis was conducted. The results suggested that CREB3L1 might exert various biological functions, especially immune modulatory functions in cancers, including the activation of innate immune response in CESC and adaptive immune response in LIHC, LUSC, PCPG (Figures 8A–D). Besides, CREB3L1 is also involved in immune response regulating signal pathway in READ, regulation of inflammatory response in UCS, interleukin 1 (IL-1) production in KICH, and DNA binding transcription activator activity in MESO (Supplementary Figure S4). In addition, KEGG analysis revealed that regulation of autophagy was the most common signaling pathway of CREB3L1 participating in multiple cancer types, including UCEC, LGG, GBM and ESCA (Figures 8E–H). Besides, chemokine signal pathway (in UCS, THCA, PCPG, LAML, and KIRP), pathway in cancer (in THYM, PCPG, and OV) and drug metabolism (in SKCM, LGG, and ACC) were also potential signaling pathways modulated by CREB3L1 in pan-cancer (Supplementary Figure S5). These results indicated that CREB3L1 might be critically involved in regulating the tumor immune microenvironment, and autophagy in diverse cancers.

### Correlation of CREB3L1 expression with TMB and MSI

**FIGURE 9:**
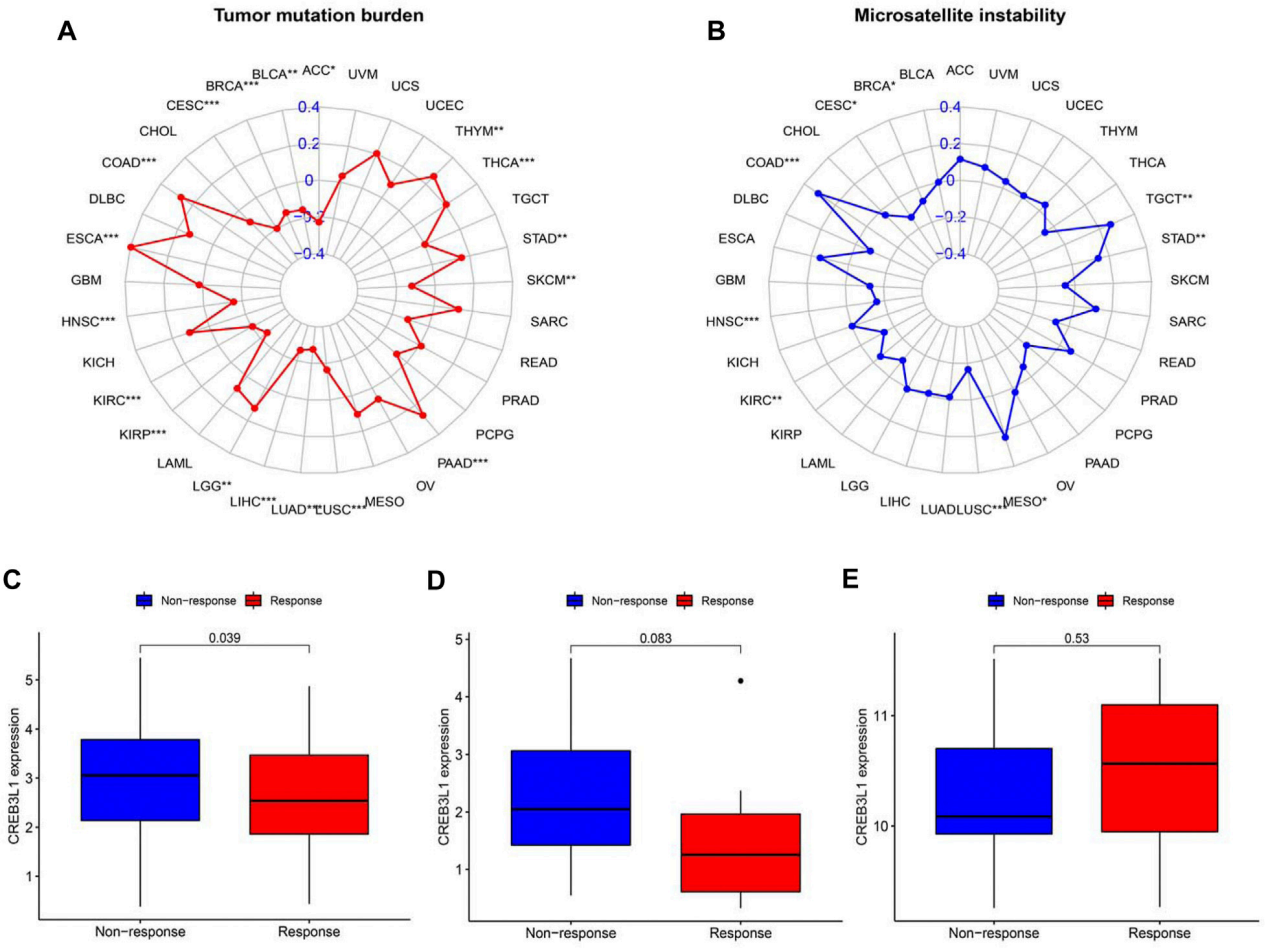
The correlation between CREB3L1 expression and TMB levels, MSI event and the immunotherapeutic efficacy. **(A)** Radar map of the relationship between CREB3L1 expression and TMB levels. **(B)** Radar map of the relationship between CREB3L1 expression and MSI event. **(C–E)** The relationship between CREB3L1 expression and the immunotherapeutic efficacy in IMvigor210 cohort **(C)**, GSE78220 **(D)**, and GSE67501 **(E)**. *p < 0.05, **p < 0.01; ***p < 0.001.

TMB and MSI have gradually emerged as biomarkers related to the immunotherapy response. Hence, we further investigated the relationship of CREB3L1 expression levels with TMB and MSI in pan-cancer, thereby speculating the potential role of CREB3L1 as a promising predictor for the immunotherapeutic efficacy in a specific cancer type. A remarkable correlation between CREB3L1 and TMB was identified in 18 cancers, a positive correlation was found in COAD, ESCA, LGG, PAAD, STAD, THCA, and THYM, while a negative correlation was suggested in ACC, BLCA, BRCA, CESC, HNSC, KIRC, KIRP, LIHC, LUAD, LUSC, and SKCM (Figure 9A). Similarly, we also found a significant association between CREB3L1 expression and MSI in nine cancers, which indicated that overexpression of CREB3L1 was positively linked to MSI in COAD, MESO, STAD, and TGCT, and was inversely correlated with MSI in BRCA, CESC, HNSC, KIRC, and LUSC (Figure 9B).

### Correlation of CREB3L1 expression with immunotherapeutic efficacy

The potential role of CREB3L1 in predicting the immunotherapeutic efficacy in cancer patients was further investigated. We extracted three GEO datasets, including GSE78220, GSE67501, and IMvigor210, to compare the differences on CREB3L1 expression levels between immunotherapy-response and immunotherapy-nonresponse patients. The results showed that CREB3L1 expression was significantly higher in non-response group in IMvigor210 cohort (*p* = 0.039) (Figure 9C), and there was a statistical tendency towards high CREB3L1 expression in non-response group in GSE78220 cohort (*p* = 0.083) (Figure 9D), whereas no close relationship between CREB3L1 expression and the immunotherapeutic efficacy was detected in GSE67501 cohort (*p* = 0.53) (Figure 9E). The results suggested that CREB3L1 might effectively predict the efficacy of PD-1/PD-LI inhibitors treatment in some specific cancer types.

### Correlation of CREB3L1 expression with drug sensitivity

**FIGURE 10:**
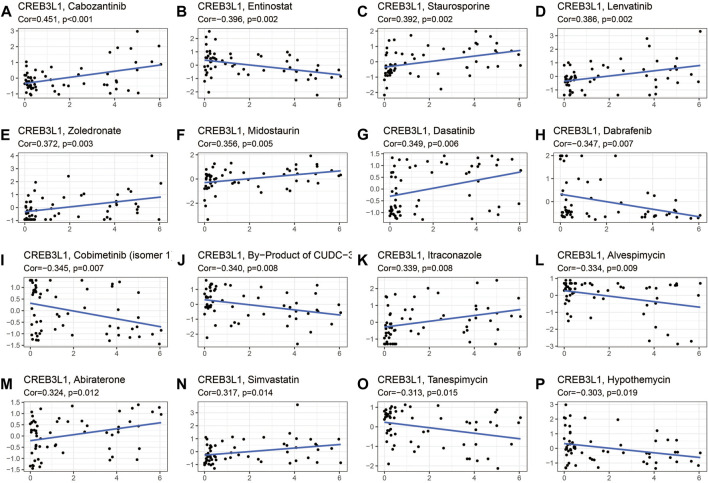
The correlation between CREB3L1 expression and drug sensitivity. The CREB3L1 was linked to the sensitivity of **(A)** cabozantinib, **(B)** entinostat, **(C)** staurosporine, **(D)** lenvatinib, **(E)** zoledronate, **(F)** midostaurin, **(G)** dasatinib, **(H)** dabrafenib, **(I)** cobimetinib (isomer1), **(J)** By-Product of CUDC-305, **(K)** itraconazole, **(L)** alvespimycin, **(M)** abiraterone, **(N)** simvastatin, **(O)** tanespimycin, and **(P)** hypothemycin.

The potential relationship between CREB3L1 expression and drug sensitivity in human cancers was also investigated (Figure 10). The results revealed that CREB3L1 was positively related to the sensitivity of several drugs, including cabozantinib (Figure 10A), staurosporine (Figure 10C), lenvatinib (Figure 10D), zoledronate (Figure 10E), midostaurin (Figure 10F), dasatinib (Figure 10G), itraconazole (Figure 10K), abiraterone (Figure 10M) and simvastatin (Figure 10N), while was negatively related to the sensitivity of entinostat (Figure 10B), dabrafenib (Figure 10H), cobimetinib (isomer1) (Figure 10I), By-Product of CUDC-305 (Figure 10J), alvespimycin (Figure 10L), tanespimycin (Figure 10O), and hypothemycin (Figure 10P). These results indicated that CREB3L1 could function as a promising predictor for the sensitivity of several anti-cancer agents, such as cabozantinib, lenvatinib, zoledronate, dasatinib, and dabrafenib, which have been commonly applied in clinical cancer management.

## Discussion

The expression pattern of CREB3L1 has been evaluated in multiple human tissues, and the biological functions of CREB3L1, such as protein secretion, cell differentiation and tissue development have been illustrated in multiple studies (Keller et al., 2018; Sampieri et al., 2019; Kamikawa et al., 2021). Recently, several studies have illustrated CREB3L1 expression and functions in different cell lineages development through conducting single-cell RNA sequencing (scRNA-seq) analysis. It has been found that FB-1 cells, a major type of fibroblast in hypertrophic scar samples have higher transcriptional activities of CREB3L1 than those in normal samples, and hypertrophic scar samples have a higher proportion of the FB-1 cells expressing CREB3L1 through investigating online scRNA-seq data, suggesting that CREB3LI may be critically involved in hypertrophic scar formation (Zhang et al., 2021a). In addition, a recent study conducting scRNA-seq analysis has revealed the critical biomarkers and process of the differentiation of fibroblasts into myofibroblasts in systemic sclerosis, and CREB3L1 may be a critical upstream transcription factor in promoting systemic sclerosis myofibroblast differentiation (Tabib et al., 2021). Notably, it has been reported that CREB3L1 expression is frequently altered in many cancers, and complex functions and mechanisms of CREB3L1 in cancer development have been clarified in several studies (Feng et al., 2017; Liu et al., 2018; Bissonnette et al., 2021; Morishita et al., 2021). Xu et al. have recently explored the cellular composition of thyroid cancer and found that CREB3L1 was upregulated in anaplastic thyroid cancer (ATC)-derived thyroid cancer cells by scRNA-seq. Mechanistically, CREB3L1 overexpression can facilitate the dedifferentiation of papillary thyroid cancer cells into ATC cells, and promote thyroid cancer progression by regulating EMT process and the mTOR signaling pathway (Luo et al., 2021). Through scRNA-seq analysis of intrahepatic cholangiocarcinoma, S100P and SPP1 are regarded as two biomarkers for two intrahepatic cholangiocarcinoma molecular subtypes. CREB3L1, which is upregulated in S100P + SPP1− intrahepatic cholangiocarcinoma tumor cells, can facilitate the invasion of S100P + SPP1− perihilar large duct type tumor cells by directly targeting S100P (Song et al., 2022). However, the investigation of CREB3L1 in human malignancies is still of absence to date, and the results of present studies are controversial. Besides, no pan-cancer analysis of CREB3L1 has been published to date. Thus, we conducted this first-time and comprehensive investigation to illustrate the roles of CREB3L1 across human cancers.

Firstly, we evaluated CREB3L1 expression pattern and its clinical value in different cancers, and the results suggested that compared with normal tissues, CREB3L1 expression levels were abnormally upregulated in several cancer types, such as BRCA, CHOL, and STAD, whereas was significantly decreased in some cancer types, such as BLCA, COAD, and LUSC. In addition, our results showed that high CREB3L1 expression was significantly associated with advanced clinical stage in several cancer types. Interestingly, our findings showed that CREB3L1 expression was abnormally decreased in BLCA, and CREB3L1 expression was increased during tumor stage progression in patients with BLCA, which seems to be contradictory. A previous study has reported that CREB3L1 was significantly downregulated in BLCA, which was consistent with our findings. CREB3L1 silenced by CREB3L1 promoter hypermethylation resulted in a more aggressive phenotype in BLCA, and no significant correlation between CREB3L1 and tumor stage was detected in this study (Rose et al., 2014). Future studies based on large-scale samples are required to illustrate the association between CREB3L1 expression and clinical stage progression in BLCA, and CREB3L1 alternations and epigenetic modifications during BLCA progression. Besides, the Kaplan–Meier survival showed that high CREB3L1 expression was significantly correlated with poor OS and DSS in KIRP, MESO, and SKCM, while was related to better OS and DSS in ACC, suggesting the prognostic significance of CREB3L1 in different human cancers. However, using OS as the endpoint of survival may decrease the utility of clinical trials, and non-cancer-induced death cannot effectively represent cancer biology. Additionally, longer follow-up time are required for OS or DSS analysis. Hence, in many clinical studies, using DFS or PFS can assess the influence of biomolecules on cancer patients more effectively. The Kaplan–Meier PFS curves showed that patients with high CREB3L1 expression had longer PFS time in ACC, PRAD and UCEC, while patients who expressed CREB3L1 highly had shorter PFS time in BLCA, KIRC and KIRP. Furthermore, the univariate Cox regression analysis results showed that CREB3L1 could serve as a protective factor in several cancer types, such as BLCA, KIRC and KIRP, while a risk factor in ACC and PRAD. These findings suggest that CREB3L1 may play different roles in cancer progression and clinical prognoses in distinct cancer types, which needs to be verified in future studies. Besides, we analyzed the characteristics of CREB3L1 mutations in pan-cancer. The results revealed that the total alteration rate of CREB3L1 was 1.4% in pan-cancer with various alternation types, such as amplification, miss mutation, and deep deletion, and patients with SKCM has the highest CREB3L1 mutation rate. To date, the biological functions of CREB3L1 mutations are mostly investigated in modulating osteogenesis process, and only few studies have reported that EWSR1-CREB3L1 fusion mutation in sarcomas (Keller et al., 2018; Bissonnette et al., 2021). The functions and mechanisms of CREB3L1 mutations are largely unknown in human neoplasms, and our investigation shed highlights on the potential roles of CREB3L1 mutations in cancer initiation and progression.

The tumor immune microenvironment fundamentally participates in cancer progression, and shows an important association with cancer patients’ survival and the therapeutic efficacy (Pitt et al., 2016). Critical components and novel targets in the tumor immune microenvironment have been uncovered that can help improve the development and application of promising therapies in cancer management, notably immunotherapies (Pitt et al., 2016; Fu et al., 2021). To uncover the potential role of CREB3L1 in modulating the tumor immune microenvironment, we firstly confirmed that CREB3L1 expression levels were positively related to immune scores and stroma scores in multiple human cancers by utilizing ESTIMATE. The crosstalk between cancer cells and immune cells has been found to contribute to the microenvironment which facilitates tumor growth and metastasis, and immune cells presenting in the tumor microenvironment play a determinative role in cancer survival and development (Hinshaw and Shevde, 2019; Marzagalli et al., 2019; Dao et al., 2022). Hence, we further explored the correlation between CREB3L1 expression levels and the abundance of infiltrating immune cells in pan-cancer. A strongly positive correlation was found between CREB3L1 expression and T regulatory cells in ESCA, and macrophages, including M0 macrophages in DLBC and LGG, M1 macrophages in KIRP, and M2 macrophages in TGCT, while a negative relationship between CREB3L1 expression and B naïve cells was detected in PAAD and TGCT, indicating that group with high CREB3L1 expression had a higher infiltrating level of immune cells, which is in line with that high CREB3L1 expression levels being connected with higher immune scores in several cancer types. Tumor-infiltrating naïve B cells have been identified as a key component of adaptive immunity with various functions, and the abundance of naïve B cells is strongly correlated with cancer patients’ prognosis (Chen et al., 2020; Downs-Canner et al., 2022). For instance, single-cell transcriptome analysis has found that the infiltrating levels of naïve-like B cells are lower in advanced NSCLC, which is closely related to worse prognosis of NSCLC patients (Chen et al., 2020). Previous studies have indicated that PAAD patients with decreasing infiltrating naïve B cell levels have worse prognose, which is in line with our results (Zhang et al., 2021b; Rajamanickam et al., 2021). These findings indicate that CREB3L1 may have regulatory effects on tumor-infiltrating naïve B cells and macrophage polarization in different cancer types, thereby influencing the cancer survival and progression. Moreover, the interaction between CREB3L1 and immune-related biomarkers was also investigated. Correlations were observed between CREB3L1 expression and several immune biomarkers such as CD28, CXCR4 and KDR in several cancers. Previous studies have found that CD28 co-stimulation is fundamental for CD8^+^ T cell rescue, and PD-1 can inhibit T cell function primarily by suppressing CD28. Moreover, CD28 co-stimulatory pathway plays key roles in modulating the tumor immune microenvironment and patients’ responses to anti–PD-L1/PD-1 therapy (Hui et al., 2017; Kamphorst et al., 2017; Duraiswamy et al., 2021). CXCR4 has been found to exert activity in modulating immune cells infiltration, such as the inhibition of cytotoxic T lymphocytes, and increase of T regulatory cells, and blocking CXCR4 can effectively improve the immunotherapeutic efficacy (Chen et al., 2019; Daniel et al., 2020). These findings suggest that CREB3L1 may regulate these crucial immune-related biomarkers to remodel the tumor immune microenvironment and influence responses to immunotherapies of cancer patients. Overall, the biological functions of CREB3L1 in modulating the tumor immune microenvironment remains a research gap, which are worth exploring in future studies.

Furthermore, the results of GSEA analysis showed that CREB3L1 was obviously involved in modulating the immune response, immune relevant pathways and chemokine release in pan-cancer, further confirming the potential roles of CREB3L1 in the tumor immune microenvironment. Besides, regulation of autophagy was identified as the most common signaling pathway modulated by CREB3L1 in pan-cancer. Previous studies have identified that CREB3L1 is an ER localized protein, and can be transported from the ER to the Golgi, which plays critical roles in modulating ER and Golgi stress responses (Sampieri et al., 2019). Notably, ER stress and Golgi apparatus have been confirmed as important triggers of autophagy in eukaryotic cells, and autophagy under ER and Golgi stress have been investigated in multiple cancers (Lin et al., 2019; Deng et al., 2020). These results suggest that CREB3L1 may function as a critical modulator of autophagy and the tumor immune microenvironment in human cancers, thereby modulating the initiation and progression of human cancers.

Previous studies have demonstrated that CREB3L1 is significantly correlated with chemoresistance in some human malignancies (Denard et al., 2012; Denard et al., 2018). In our study, we confirmed that CREB3L1 is significantly correlated with the sensitivity of several anti-cancer drugs, especially targeted therapy agents, such as cabozantinib, lenvatinib, and dabrafenib. These findings indicate that CREB3L1 exhibits great potential as a predictive biomarker for anti-cancer drugs sensitivity and a novel therapeutic target in human cancers, and dynamic monitoring of CREB3L1 expression may be a valuable approach to effectively evaluate therapeutic responses of cancer patients, thus helping choose the most suitable treatment strategy for the individual patient. However, the mechanisms linked to the involvement of CREB3L1 in modulating drug sensitivity are still inconclusive, which may be a future direction for further research on CREB3L1. Furthermore, there is no related research on the correlation between CREB3L1 expression and the immunotherapy efficacy until now. Hence, we deduced that CREB3L1 may play critical roles in cancer immunotherapies, and influence the immunotherapeutic efficacy. TMB and MSI have been identified as critical regulators that affect the effectiveness of immune checkpoint inhibitors in the treatment of several tumors (Boland and Goel, 2010; Samstein et al., 2019). Higher TMB levels and MSI levels are generally acknowledged as important biomarkers related to the high therapeutic efficacy in multiple immune checkpoint inhibitors treated cancers (Boland and Goel, 2010; Samstein et al., 2019). Our study presented evidence for the possible relationship of CREB3L1 expression with MSI and TMB in different cancers. Our results showed that CREB3L1 expression was positively correlated with TMB and MSI in COAD and STAD, indicating that patients with COAD or STAD who express CREB3L1 highly may have better responses to immune checkpoint inhibitors, while a negative connection of CREB3L1 with TMB and MSI was detected in BRCA, CESC, HNSC, KIRC, and LUSC. These findings indicate that CREB3L1 may function as an effective predictor for the efficacy of immune checkpoint inhibitors treatment in several cancers, such as BRCA, COAD and STAD. Therapeutic antibodies that block the PD-1/PD-L1 pathway have shown efficacy and have been approved in multiple cancers (Yi et al., 2022). However, the responses of the majority of patients to PD-1/PD-L1 inhibitors are limited, and previous studies have suggested that PD-1/PD-L1 expression, MSI and TMB levels are not robust biomarkers for predicting the efficacy of immune checkpoint inhibitors. Developing novel predictive biomarkers for PD-1/PD-L1 inhibitors therefore plays a key role in maximizing the immunotherapeutic efficacy (Zeng et al., 2019; Yi et al., 2022). To further confirm the potential role of CREB3L1 as the predictor for the immunotherapeutic responses, immunotherapy-related cohorts were enrolled in our investigation. We found that patients with no-response to PD-1/PD-L1 inhibitors have a higher CREB3L1 expression in patients with urothelial carcinoma and renal cell carcinoma, indicating that CREB3L1 is a promising predictor of the anti-PD-1/PD-L1 treatment benefits, thereby providing a new biomarker for predicting the immunotherapeutic efficacy.

However, there are some limitations in this quality study. The data enrolled in our study is extracted from online databases instead of our own clinical samples. Thus, these findings in our present study should be further verified by our own patients. In addition, the roles of CREB3L1 in a specific cancer type remain unclear, and the mechanisms by which CREB3L1 modulates the tumor immune microenvironment are still unknown. Future studies are needed to verify these findings in experiments.

In conclusion, we performed a comprehensive pan-cancer analysis of CREB3L1 by assessing multifaced aspects of CREB3L1, including the expression pattern, prognostic significance, genetic mutations, and immune-related signature, revealing the promising role of CREB3L1 as a promising indicator for the prognosis and immunotherapeutic efficacy of cancer patients and its immunoregulatory effects in pan-cancer. Our research provides highlights into the biological functions of CREB3L1 in pan-cancer, and the candidate effect of CREB3L1 in predicting the prognosis and immune-related phenotypes.

## Data Availability

The original contributions presented in the study are included in the article/Supplementary Material, further inquiries can be directed to the corresponding authors.
