# Supplementary figures and images for: Pan-cancer analysis of CREB3L1 as biomarker in the prediction of prognosis and immunotherapeutic efficacy
Source: Front Genet. 2022 Sep 9;13:938510. doi: 10.3389/fgene.2022.938510 (PMC9511413; doi:10.3389/fgene.2022.938510)

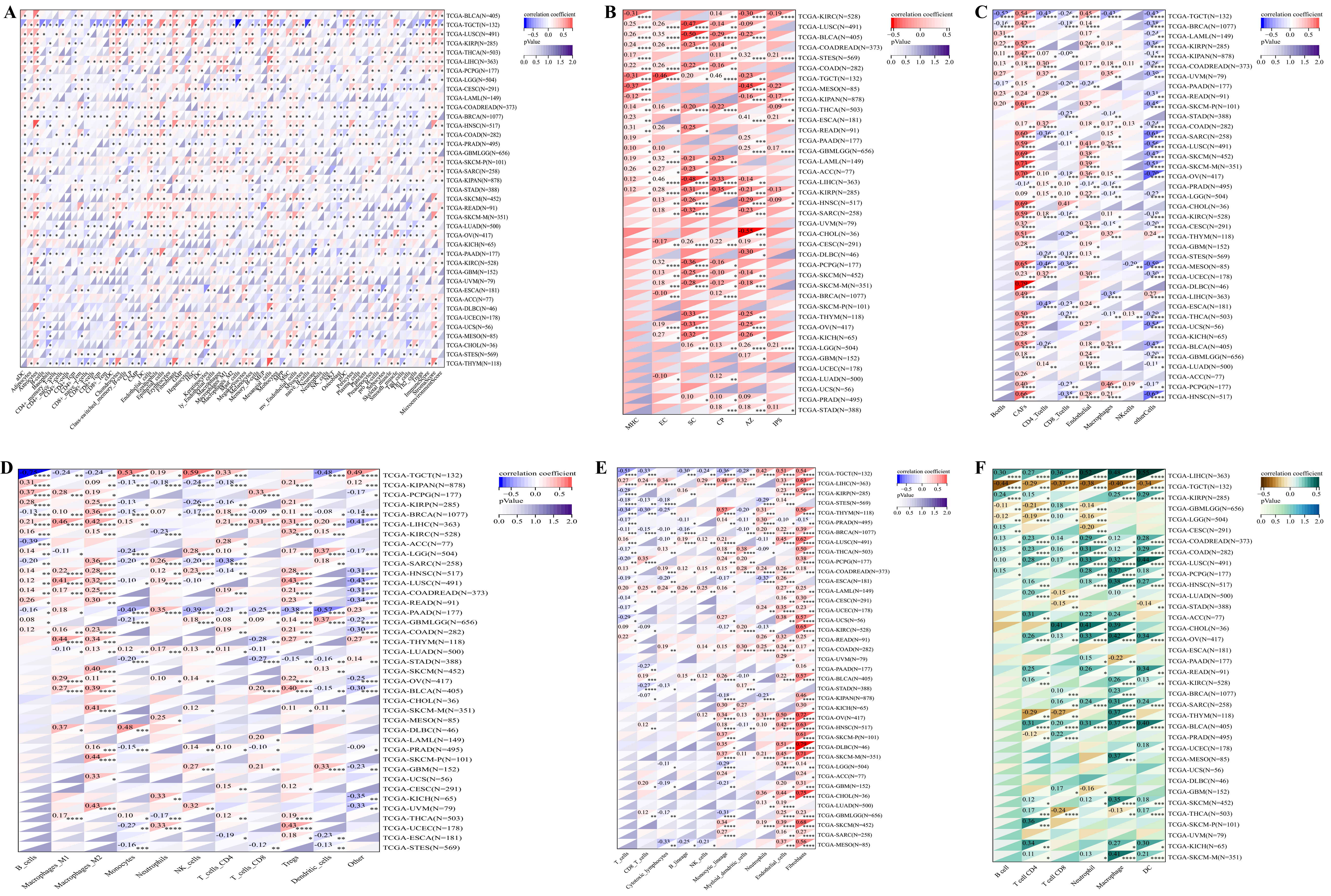

Supplement: Supplementary file 1 [file Image3.JPEG]

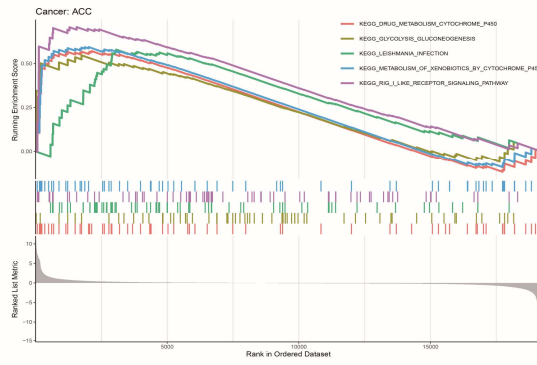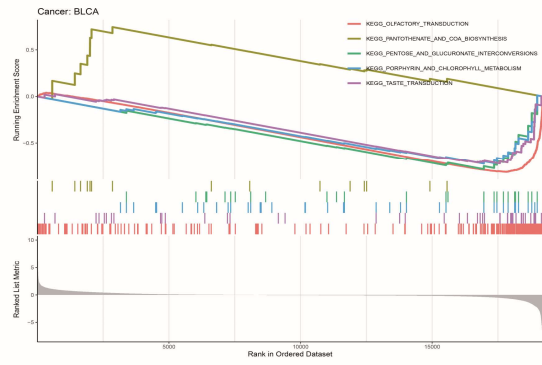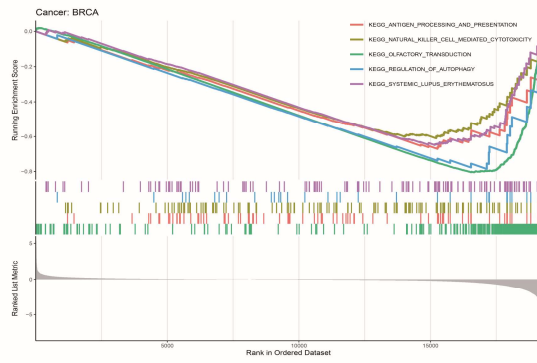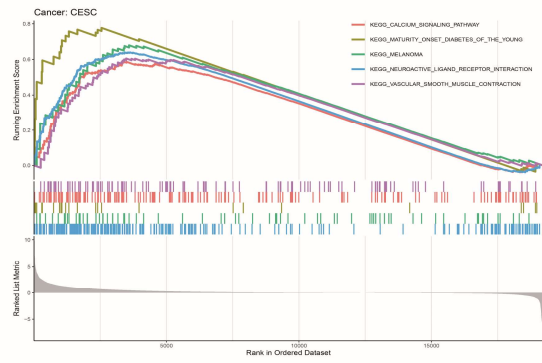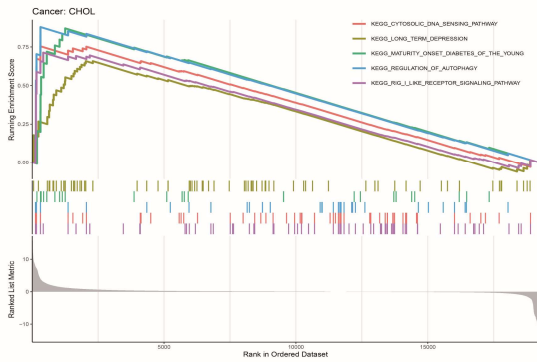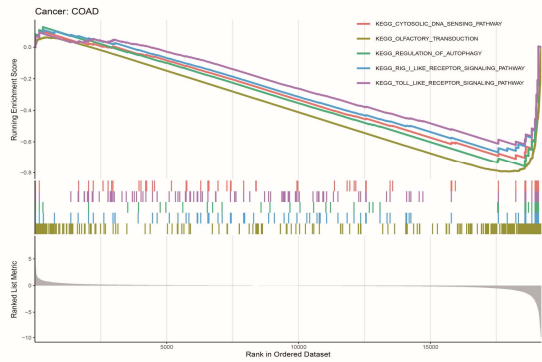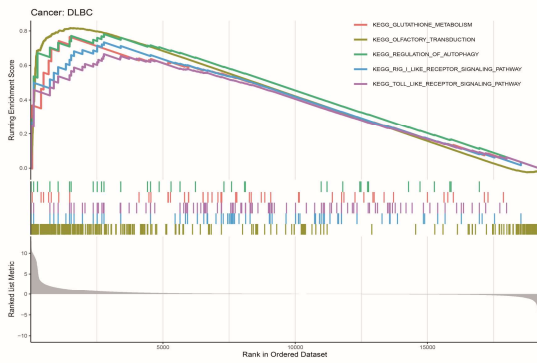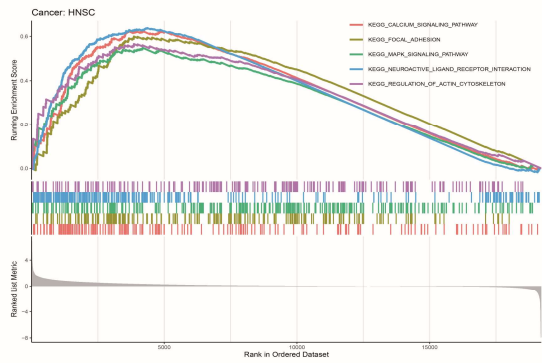

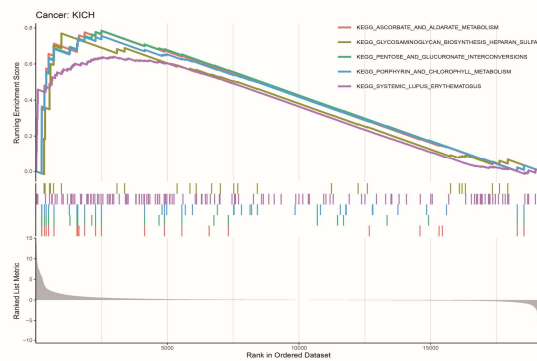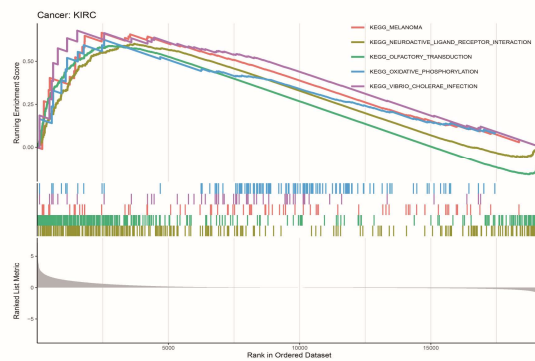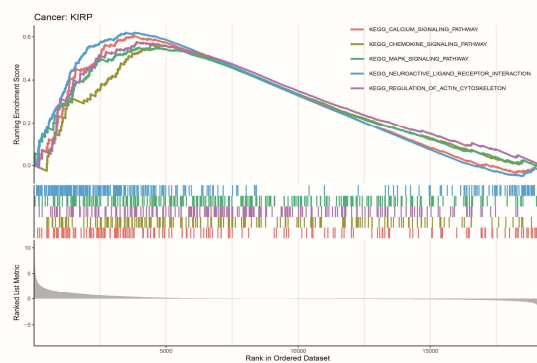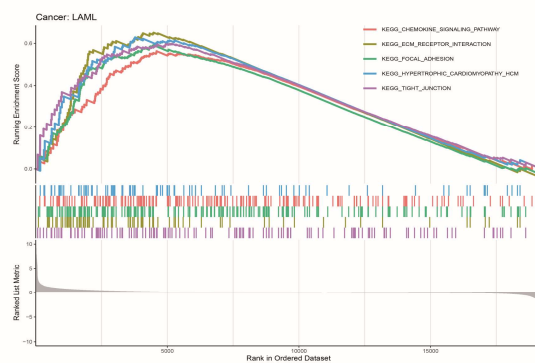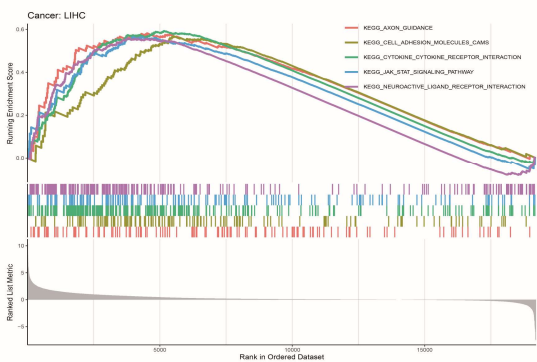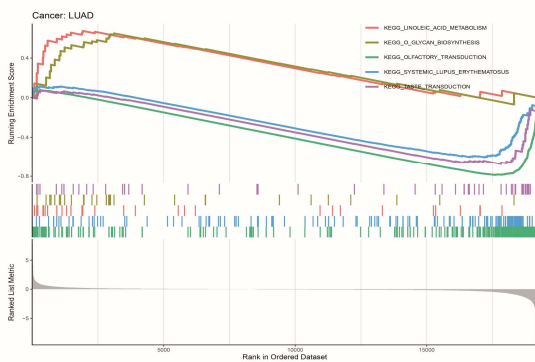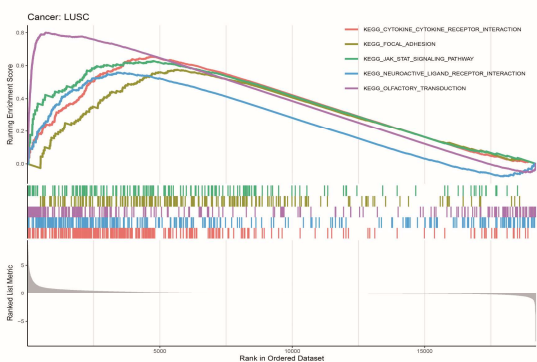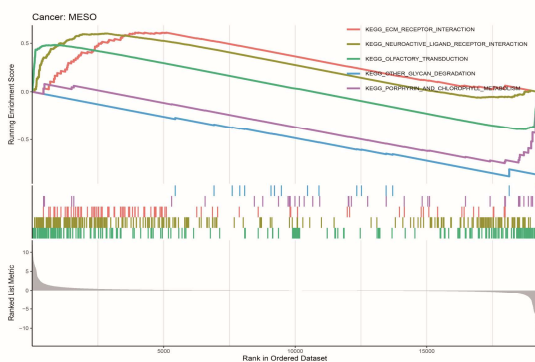

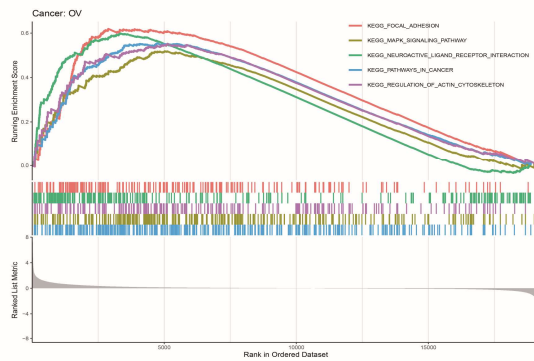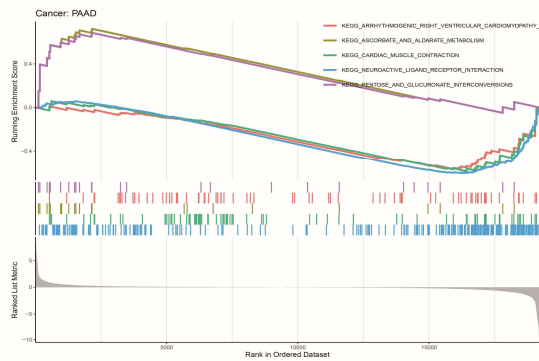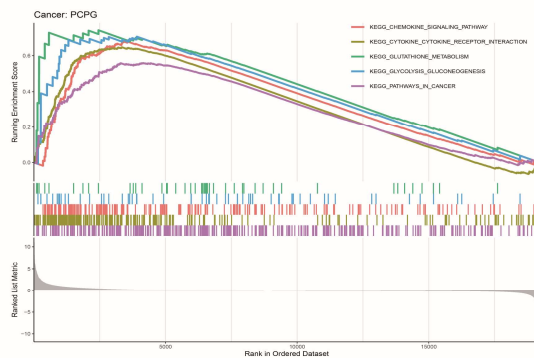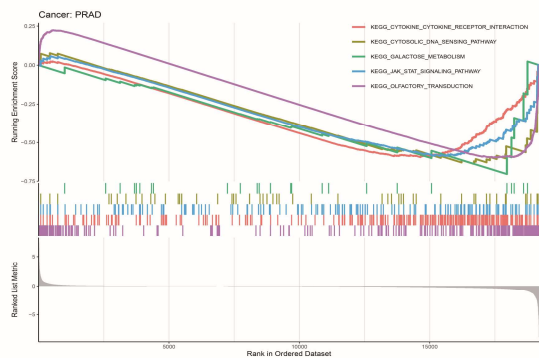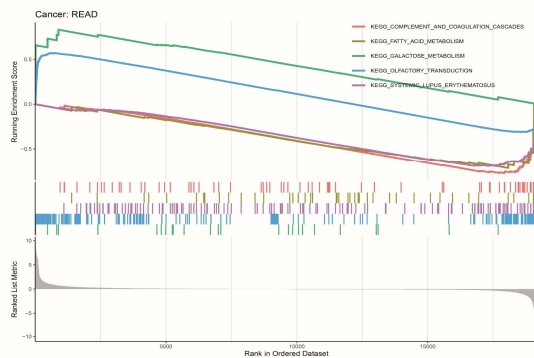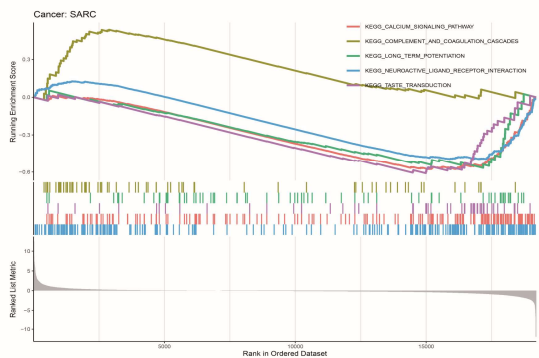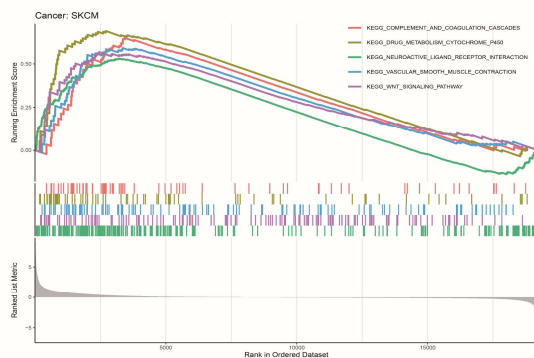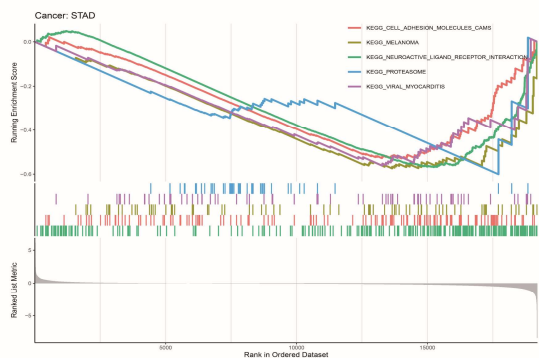

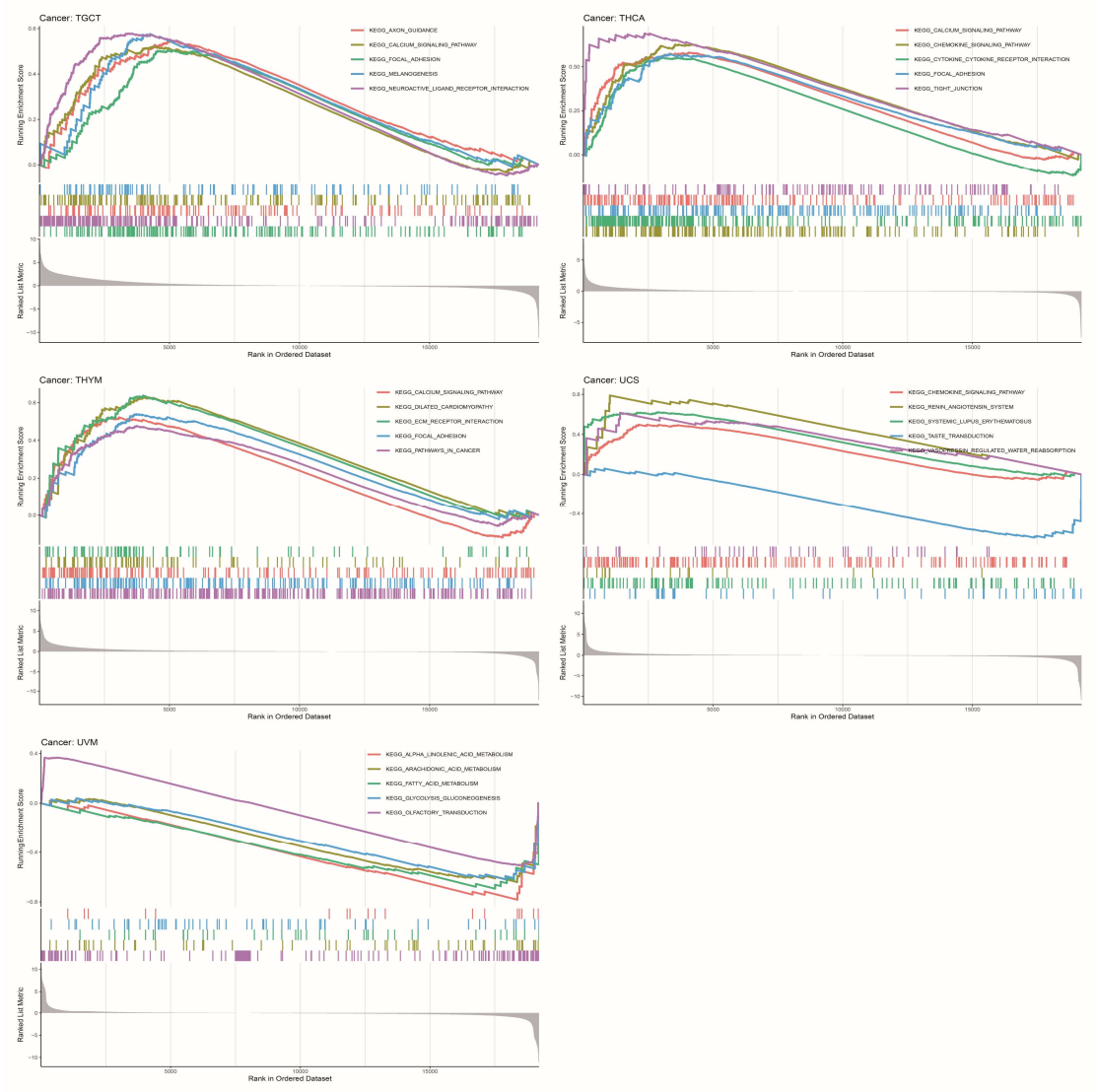

Supplement: Supplementary file 2 [file Image5.PDF]

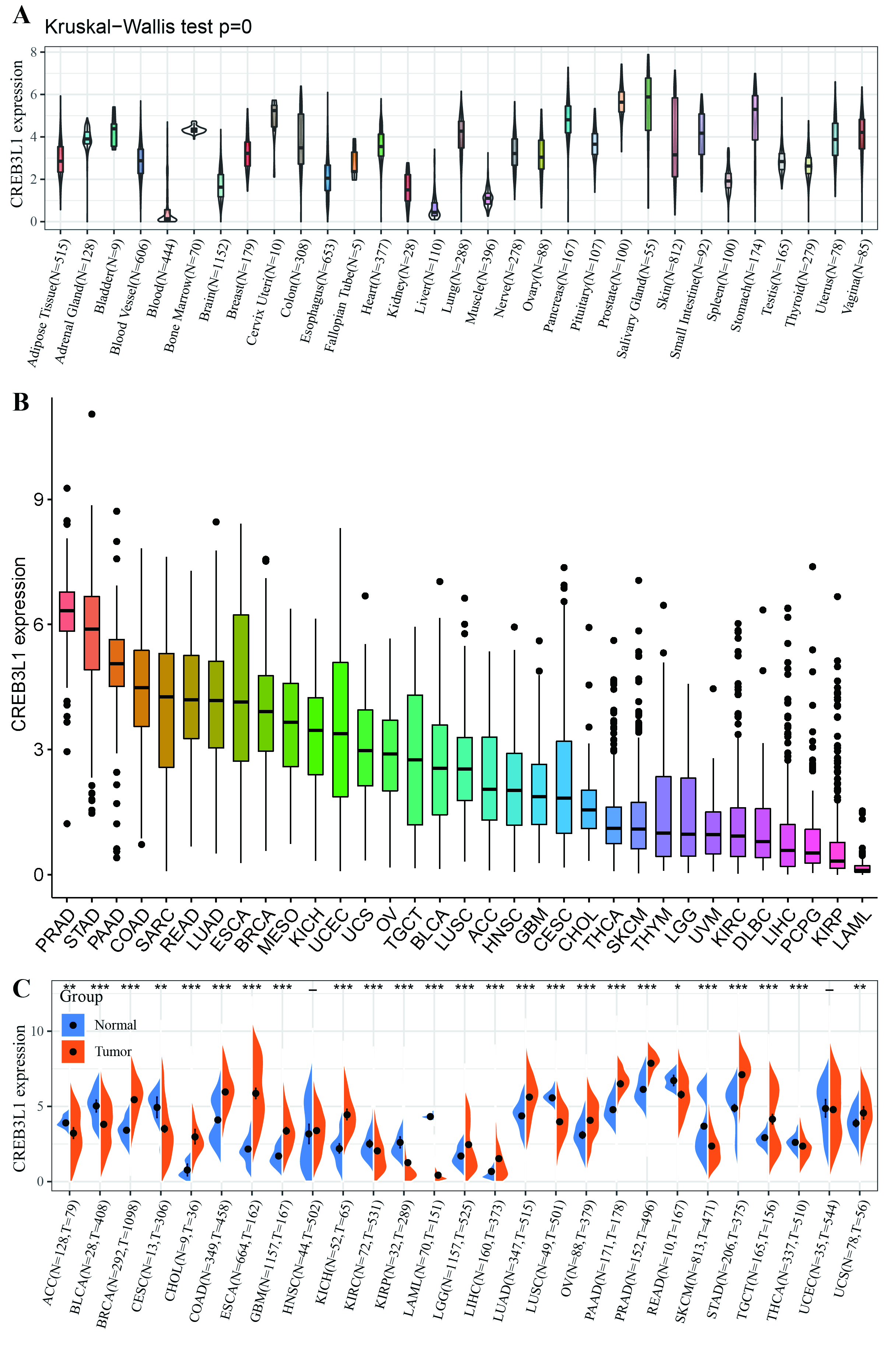

Supplement: Supplementary file 3 [file Image1.JPEG]

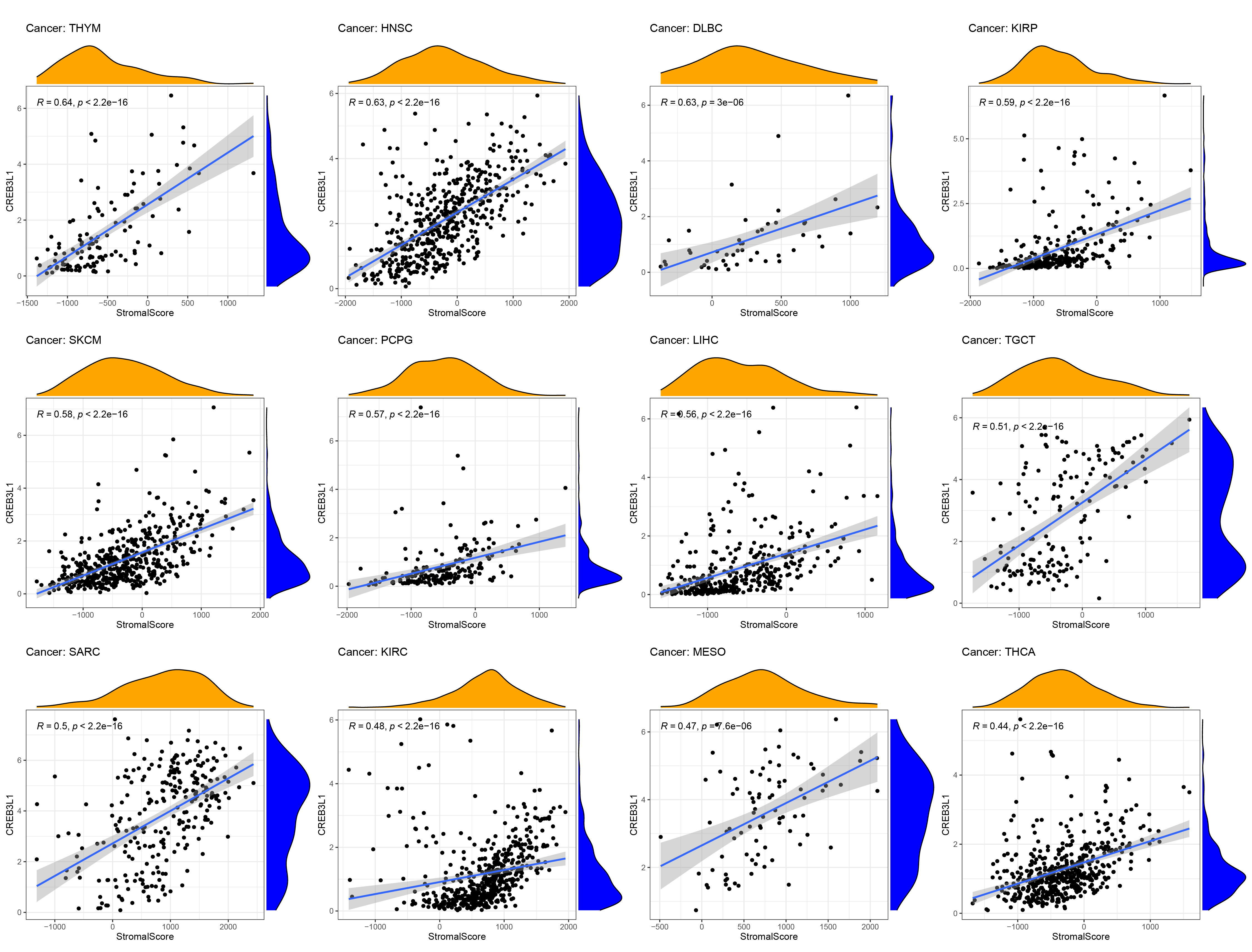

Supplement: Supplementary file 4 [file Image2.JPEG]

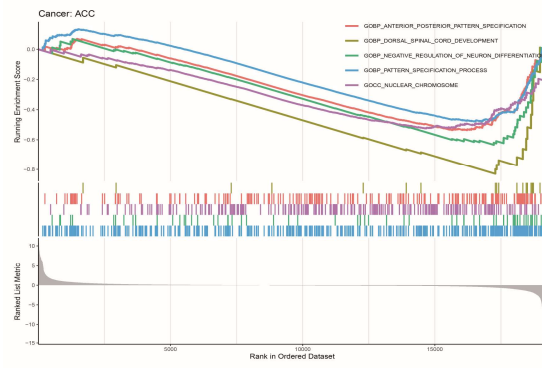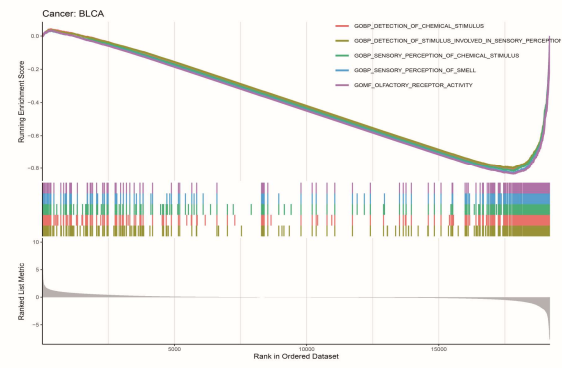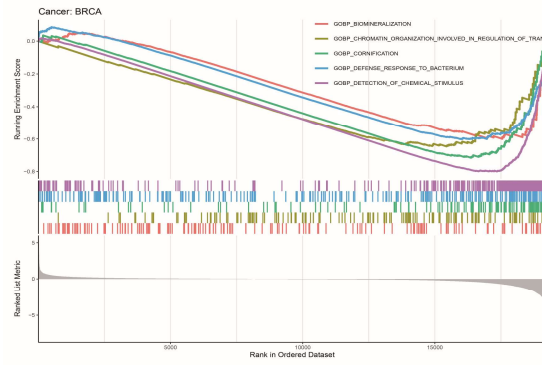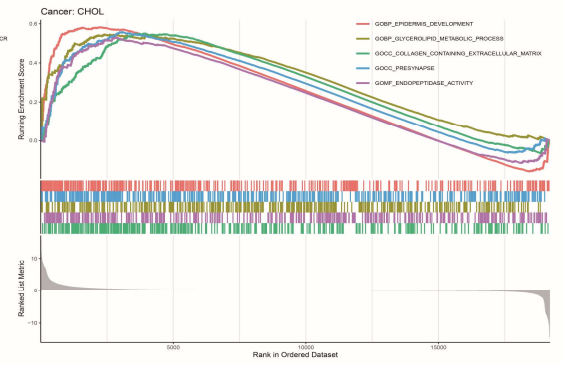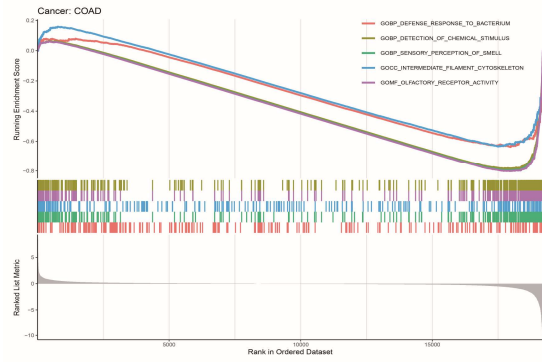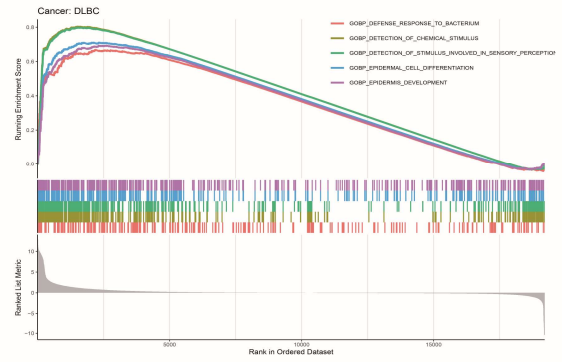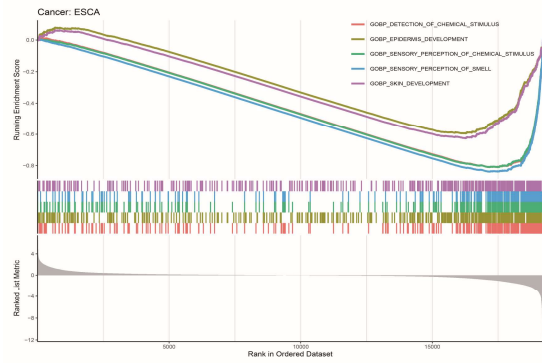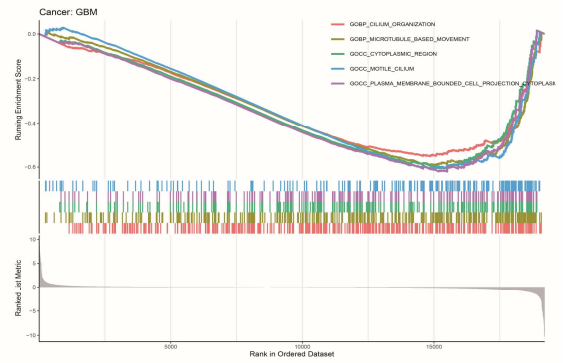

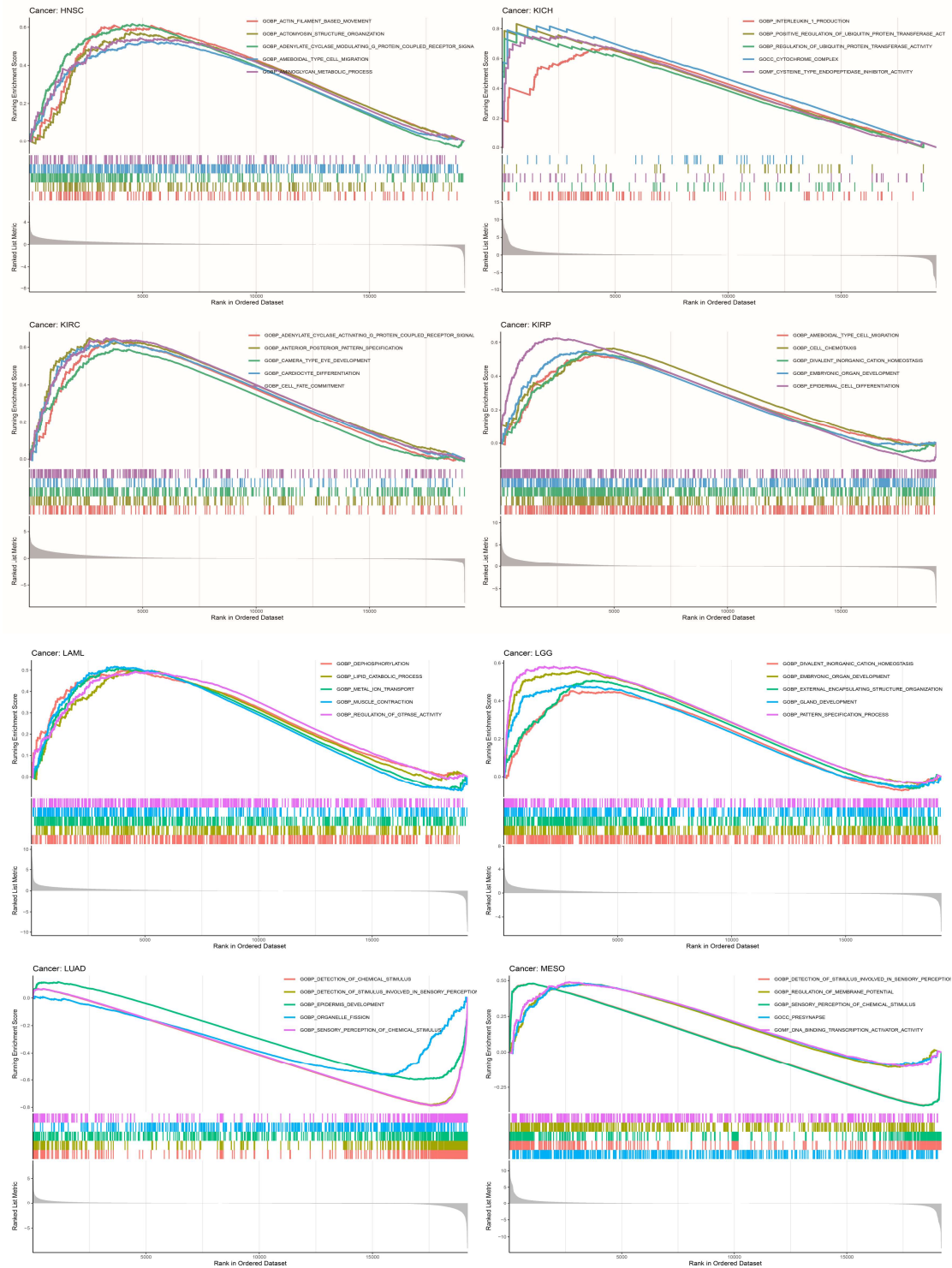

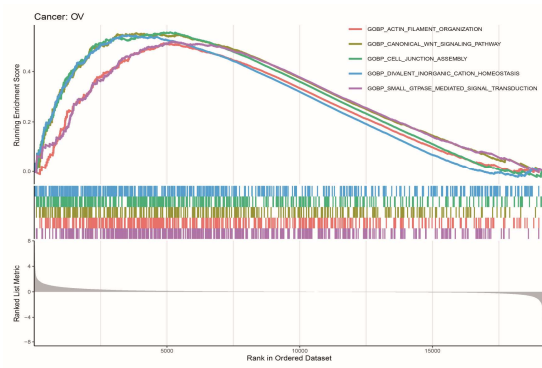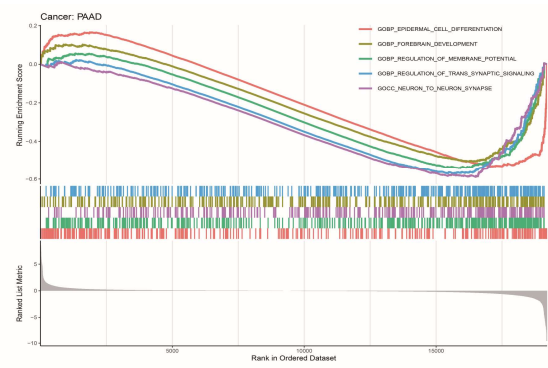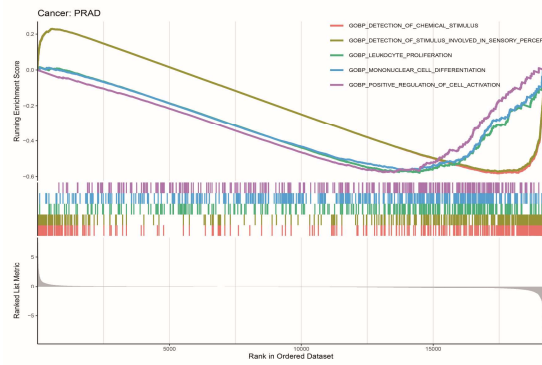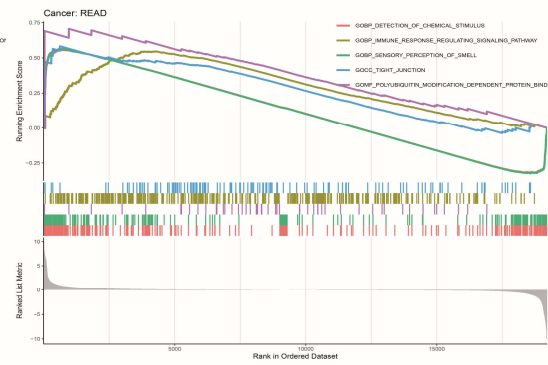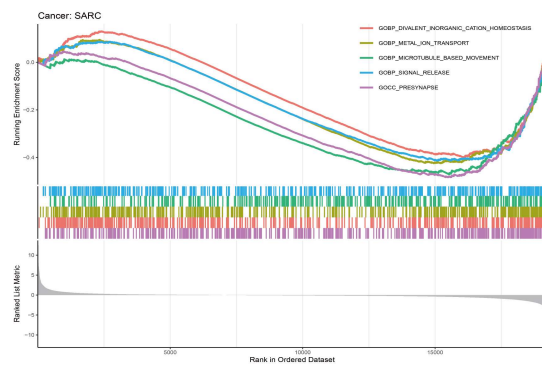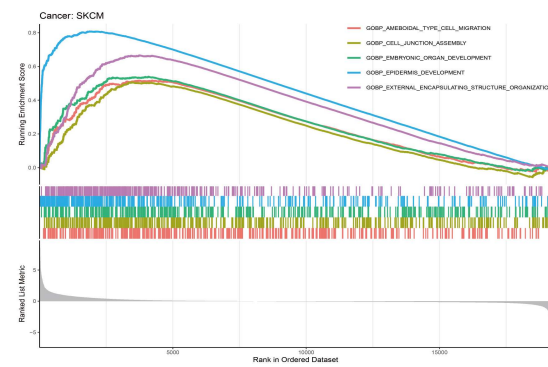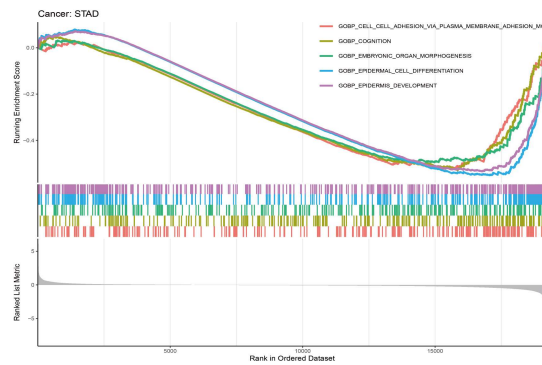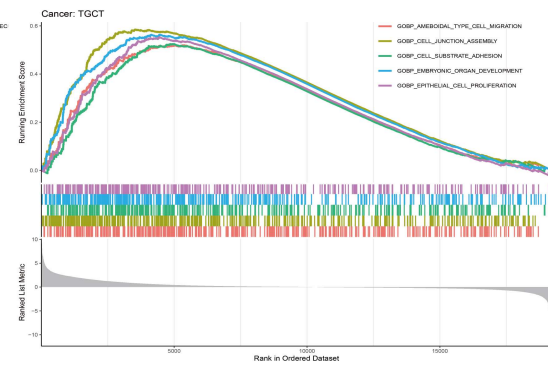

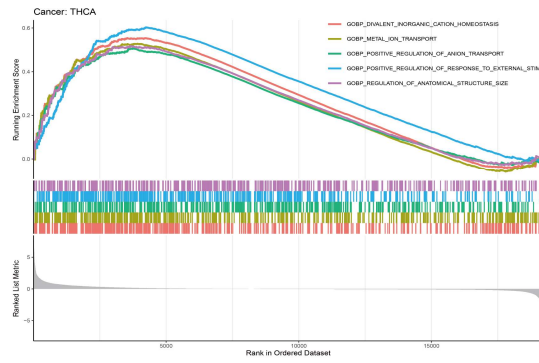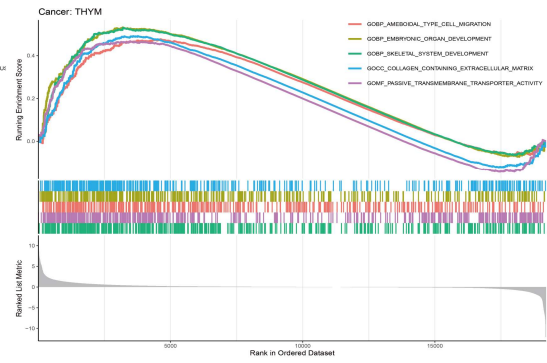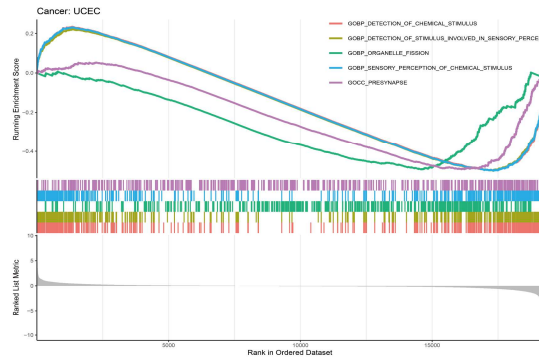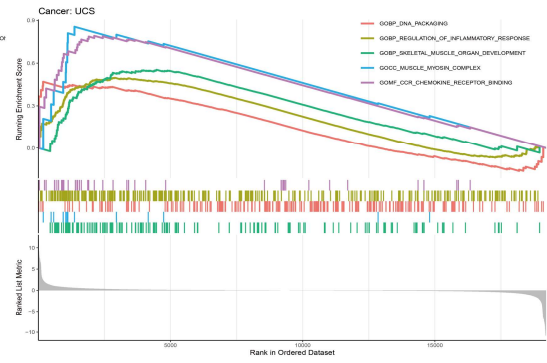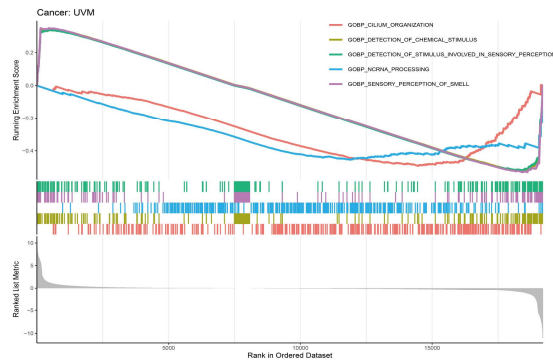

Supplement: Supplementary file 5 [file Image4.pdf]
