# Supplementary material for: Pan-cancer analysis of CREB3L1 as biomarker in the prediction of prognosis and immunotherapeutic efficacy
Source: Front Genet. 2022 Sep 9;13:938510. doi: 10.3389/fgene.2022.938510 (PMC9511413; doi:10.3389/fgene.2022.938510)
Supplement: Supplementary file 6 [file DataSheet2.DOCX]

**Supplementary Figure Legends**

**Figure S1.** The expression pattern of CREB3L1. (A) CREB3L1 expression in normal tissues from GTEx database. (B) CREB3L1 expression in 33 cancer types from TCGA database. (C) Comparison of CREB3L1 mRNA expression between cancer and matched normal samples in 27 cancers from GTEx database. *P < 0.05, **P < 0.01, ***P < 0.001.

**Figure S2.** The correlation between CREB3L1 expression and stromal score in pan-cancer.

**Figure S3.** Correlation between CREB3L1 and immune cell infiltration in (A) XCELL, (B) IPS, (C) EPIC, (D) QUANTISEQ, (E) MCPcounter, and (F) TIMER database.

**Figure S4.** GO functional annotation of CREB3L1 by GSEA analysis based on the TCGA.

**Figure S5.** KEGG pathway analysis of CREB3L1 by GSEA analysis based on the TCGA.
