# Supplementary material for: Pan-cancer analysis of CREB3L1 as biomarker in the prediction of prognosis and immunotherapeutic efficacy
Source: Front Genet. 2022 Sep 9;13:938510. doi: 10.3389/fgene.2022.938510 (PMC9511413; doi:10.3389/fgene.2022.938510)
Supplement: Supplementary file 7 [file DataSheet1.docx]

**The links for raw data obtained from publicly available datasets in this article as follow:**

1. TCGA-pan-cancer project: https://xena.ucsc.edu/

2.CCLE database: https://portals.broadinstitute.org/ccle/about

3. http://www.sangerbox.com/tool

4. TISIDB database: http://cis.hku.hk/TISIDB/index.php

5. https://www.gsea-msigdb.org/gsea/downloads.jsp

6. https://tcga.xenahubs.net

7. GSE78220, GSE67501, and IMvigor210 projects: https://www.ncbi.nlm. nih.gov/geo/

8. CallMiner database: https://discover.nci.nih.gov/cellminer/home.do

Mutation data and survival data of TCGA pan-cancer can be required on https://www.jianguoyun.com/p/DT9AGIsQhYLGChjcxb0EIAA and https://www.jianguoyun.com/p/Dd72QDkQhYLGChi34b0EIAA.
